# Digital Innovations for Clinical Assessment in Acquired Brain Injury: Scoping Review

**DOI:** 10.2196/73331

**Published:** 2025-11-05

**Authors:** Carl O'Brien, Aoife Murray, Gerard McManus, Chantel Debert

**Affiliations:** 1 Royal College of Physicians of Ireland Dublin Ireland; 2 Hotchkiss Brain institute University of Calgary Calgary, AB Canada; 3 School of Medicine University College Dublin Dublin Ireland; 4 Department of Clinical Neuroscience University of Calgary Calgary, AB Canada

**Keywords:** acquired brain injury, digital health technology, clinical assessment, scoping review, eHealth

## Abstract

**Background:**

Acquired brain injury (ABI) is a leading global cause of morbidity, affecting millions, many of whom face a diverse range of cognitive, physical, and psychological challenges, often made worse due to limited access to timely assessment and appropriate care. In recent years, digital technologies have emerged as potential tools to support more accessible, efficient, and scalable methods for assessment; however, the breadth of research in this area remains unclear.

**Objective:**

This scoping review aimed to identify and synthesize contemporary research on how digital technologies may help screen, assess, and monitor the complications of ABI in order to uncover trends, themes, and priorities for future research.

**Methods:**

Following the Arksey and O’Malley framework and PRISMA-ScR (Preferred Reporting Items for Systematic Reviews and Meta-Analyses extension for Scoping Reviews) guidelines, a systematic search was conducted across Embase, MEDLINE, and Scopus, as well as four clinical trial registries to help capture gray literature. A search string incorporating terms related to “ABI,” “clinical assessment,” and “digital tools” was developed a priori. Studies from 2013 to 2024 leveraging digital health tools, for example, smartphones, tablets, websites, telemedicine, and virtual reality, to aid ABI complication assessment were included. Exclusion criteria comprised studies involving bespoke clinical hardware (eg, radiographs and EEG monitors), nonhuman subjects, or review articles. Following this, data synthesis and domain mapping were performed.

**Results:**

Of 5293 screened records, 88 met the inclusion criteria: 2 retrospective studies, 4 qualitative studies, 35 cohort studies, 42 cross-sectional studies, and 5 randomized controlled trials. The median sample included 26 participants with ABI; 51 studies also involved non-ABI participants (median of 10 participants included). Digital platforms varied, with 45 studies using smartphone or tablet technologies, 23 PC or web-based platforms, 11 telemedicine solutions, and 9 virtual reality platforms. The predominant research themes included the use of digital technology to aid screening for traumatic brain injury, identifying or monitoring symptoms or functional outcomes; physical examination, the assessment of cognition and communication, and providing a comprehensive consultation. Most tools were reported to be well-tolerated, with accuracy often described as comparable to standard assessments. However, the studies were heterogeneous, with limited validation of tools across broad representative populations or multiple sites or studies. There was also little discussion on potential ethical concerns such as accessibility, access, and data privacy.

**Conclusions:**

This investigation provides an extensive overview of current research trends and highlights the need for larger, more rigorous studies to optimize the use of digital technologies in ABI assessment, as well as gaps in the assessment of common complications. Expanding research into underexplored ABI complications, broadening the scope of assessments to include a broader range of complications, and including larger, more diverse populations will be critical for advancing the field and improving outcomes for individuals with ABI.

## Introduction

### Background

Acquired brain injury (ABI), either due to traumatic brain injury (TBI), neoplasm, encephalitis, or a myriad of other causes, is a condition with an impact on millions of individuals worldwide [1]. In many developed countries, there has been a surge in survival rates thanks to advances in acute care and neurosurgery [2]. These have not been mirrored by a corresponding enhancement in equitable access to quality care during the subacute and chronic phase postinjury [3,4]. Though survival may be improving, the burden of morbidity and disability continues to rise [5]. There is a wide range of potential complications from ABI, including pain, cognitive deficits, psychological distress, mobility, sensory, and balance issues [6]. Often, these issues are hard to screen for and manage in the acute setting [7]. Accessing appropriate care to identify, treat, and diagnose the complications of ABI can feel like an impossible task for individuals and their families [8]. Most health authorities lack an overarching strategy to address increasing demands, with limited services in hospitals or the community, threatening to overwhelm health systems [1,9].

With the advent and acceleration of digital technologies, health care professionals caring for individuals with ABI have an opportunity to use innovative techniques to assess, monitor, and manage complications [10]. The proliferation of mobile phone apps and the ubiquity of social media have made people with ABI and caregivers more connected than ever, even in the most remote environments [11]. Health professionals have also seen an enormous, if fragmentary, increase in digital tools in their practice, although most are not specific to ABI care [10]. Innovations in virtual reality (VR), augmented reality, wearable technology, and artificial intelligence have the potential to revolutionize the nature and administration of ABI and make it more equitable, effective, and personalized.

Research into digital technology in health care has accelerated in recent years, including in the area of ABI assessment, but there is little information on the breadth of research in this space [12]. It is vital to map the advances in ABI assessment to inform strategies for overall care, and reduce the burden on patients, families, and health services.

### Objectives

This study aimed to identify and chart research on how digital innovations may assist with ABI assessment and symptom monitoring. The primary objective was to describe research in the use of ubiquitous “off-the-shelf” digital technologies (eg, smartphones, tablet computers, websites, VR platforms, and telemedicine platforms) to screen for, assess, and monitor the complications of ABI in the past decade. The secondary objective was to chart the predominant technologies being studied, how technology use has changed over time, the characteristics of the study participants, and the predominant study settings, methodologies, outcome measures, and findings.

## Methods

### Overview

This scoping review was conducted in accordance with the framework for scoping reviews outlined by Arksey and O’Malley [13] and refined by Levac et al [14]. This structure was selected to facilitate a comprehensive and descriptive mapping of the rapidly evolving research landscape related to the use of digital technologies for clinical assessment in individuals with ABI. The framework outlines five key steps in developing and carrying out a scoping review: (1) identifying the research question; (2) identifying relevant studies; (3) developing criteria for appropriate study collection; (4) charting the data; and (5) collating, summarizing, and reporting the results, with or without expert consultation. An optional sixth step is the inclusion of an appraisal of quality, which may be appropriate depending on the research question. The review adhered to the PRISMA-ScR (Preferred Reporting Items for Systematic Reviews and Meta-Analyses extension for Scoping Reviews) checklist, which provides best practice advice for presenting the search strategy, findings, and implications [15] (Multimedia Appendix 1). This approach mirrors that taken in other scoping reviews on similar topics in depression, aging, and cancer rehabilitation [16-18].

### Identifying the Research Question

The research question was developed by all authors through an iterative process. A preliminary search was carried out by the lead author on Medline to provide an approximate outline of the breadth and nature of studies on digital tools for ABI Assessment.

The overarching objective of the scoping review was to identify what English language studies exist from the past 10 years that evaluate the use of digital technologies to screen for an ABI, or assess for or monitor the complications of ABI.

We specifically evaluated:

1.1 The predominant technology platforms and apps being evaluated.

1.2 How the type and frequency of technology platform use changed over time, for example, smartphone/tablet, computer/web-based, or telemedicine platforms.

1.3 The predominant themes of ABI assessment for which tools were being developed to support, for example, cognitive assessment and physical examination.

1.4 The participant cohorts included, for example, demographic features, disease severity, and underlying etiology.

1.5 The characteristics of included studies, that is, what are the common themes in the studies’ aims, methodologies, outcome measurements, and main findings.

### Identifying Relevant Studies

Prior to developing a search string, definitions for the key concepts of the study were agreed upon. ABI was defined as any individual who sustained an injury to the brain that was not developmental in nature or acquired at birth [19]. Due to the unique presentation and specific needs of individuals with stroke [20], it was agreed not to include this cohort in the scoping review. Digital health technologies were defined in accordance with the US Food and Drug Administration as encompassing mobile health, health IT, wearable devices, telemedicine, and AI-assisted tools for health care assessment and monitoring [21]. Assessments were defined as any method to screen for, evaluate, quantify, or monitor ABI or its complications. The context was defined as any digital technology being used to aid assessment, monitoring, or screening tests on individuals with ABI in any health setting.

A systematic search strategy was developed a priori in consultation with a research librarian. The search was applied across MEDLINE (via Ovid), Embase (via Ovid), and Scopus. A comprehensive search string was developed by combining controlled vocabulary (eg, MeSH [Medical Subject Headings] terms) and free-text terms related to three core concepts: (1) ABI (eg, “acquired brain injury,” “traumatic brain injury,” “brain tumour,” and “encephalitis”), (2) digital technologies (eg, “telemedicine,” “mobile health,” “eHealth,” “apps,” “wearables,” and “artificial intelligence”), and (3) clinical processes (eg, “assessment,” “screening,” “monitoring,” and “measurement”). Boolean operators (“AND” and “OR”) and truncation were used to combine synonyms and refine results. Each database-specific syntax was adapted to its indexing system. The development process included iterative testing and refinement of the search terms and the review of reference lists of included studies to identify additional keywords. The full final search strategy for each database is available in Multimedia Appendix 2 [16-28].

A strategy for including gray literature was also confirmed A Priori. Scopus was used for its innovative feature that searches across multiple preprint databases, Embase’s feature for screening for conference abstracts was implemented, which was then used to search for full-text articles, both published and preprints. As well as research databases, 4 clinical trials registries were searched using broad search terms: the International Clinical Trials Registry, ClinicalTrials.gov, the European Union Clinical trials register, and the UK’s ISTRCN (originally called International Standard Randomized Controlled Trial Number).

### Developing Criteria for Appropriate Sources of Evidence

The criteria for including articles in the process of charting and data mapping were decided by all authors following the preliminary search. We used a broad inclusion strategy to ensure the capture of exploratory, pilot, and feasibility work in addition to more established technologies and tools. Studies were deemed to be clinically relevant if they included individuals with ABI in their evaluations and included a description of the outcomes, regardless of the size of the study or the nature/quality of the study design.

The inclusion criteria were all studies that:

1. Were published in English (the language of the study authors).
2. Focused on digital health tools [23,29] for screening, evaluating, or monitoring ABI symptoms (either solely or as part of a larger patient cohort).
3. Reported primary research findings.
4. Were published between January 2013 and December 2024 inclusive. The decision to limit the search to this timeframe was informed by a preliminary search, which found that digital technologies predating 2013 were largely obsolete or incompatible with current digital platforms and clinical standards [22,23]. This approach has precedent in similar digital health scoping reviews [16-18].

The exclusion criteria were studies that:

1. Did not specifically address ABI or solely focus on participants with stroke.
2. Focused on nondigital methods of assessment without integrating digital tools, or when digital tools were not the focus of the evaluation.
3. Were narrative or systematic reviews, meta-analyses, editorials, or opinion pieces lacking original research data.
4. Were descriptive, protocols only.
5. Were still in development, that is, they did not involve assessment of real human participants with ABI.
6. Did not have accessible full texts.
7. Involved bespoke clinical devices that were not off-the-shelf or readily available digital technologies as defined by the US Food and Drug Administration and WHO guidelines on digital health and consumer-grade devices [21,23]. Examples of technologies and tools that do not fit these definitions could include digital intracranial pressure monitors, digital radiology devices such as MRI scanners, electroencephalograms, robotic gait aids, digital force plates, etc.

All articles captured in the systematic search were saved on a systematic review platform, Rayyan (Rayyan Systems Inc). The titles and abstracts of all articles were screened for relevancy by 2 authors working independently and blinded to each other’s decisions, with a third tie-break author included if needed. Following this, 2 authors reviewed all full-text articles for final inclusion based on prespecified criteria. Discrepancies were discussed and resolved through consensus; where consensus was not reached, a third author acted as an adjudicator.

### Charting the Data

The Arksey and O’Malley framework [13], and subsequent refinements by Levac et al [14] and PRISMA-ScR [15], recommend that a plan for the collection of data from included reports (ie, data charting), be developed and refined iteratively by the research team to ensure consistency and relevance to the research question. Using this approach, the research team carried out a preliminary search and identified the following key data elements to include: first author, year of publication, nature of the technology, target domain for assessment (eg, cognition, physical findings, and emotion/behavior), study aim, participant diagnosis and demographics, site and setting of the research, study methodology, primary outcome measures, and key findings including summary statistics. These data elements were agreed upon iteratively, following the preliminary and subsequent searches, to provide a comprehensive overview of the scope of research investigating digital technologies in ABI assessment, and to illustrate the breadth and variety of study design, setting, cohort characteristics, and quality. Data charting was completed by one author.

### Collating, Summarizing, and Reporting the Results

Consistent with stage 5 of the Arksey and O’Malley framework [13], and subsequent refinements [14-16], the charted data were collated, summarized, and reported using a combination of narrative description, descriptive numerical summary, and thematic analysis. The results were presented in narrative form, supported by a comprehensive data table providing a synthesis of setting and cohort characteristics.

A thematic analysis was developed iteratively by the study authors, where the type of clinical assessment served as the central organizing theme. Studies within these thematic domains were systematically compared by population, technology platform and specific app, study design, population, outcome measure, and findings—this was presented using a combination of narrative description and data tables.

### Evaluation of Study Quality

The Arksey and O’Malley guidelines advise that a formal quality appraisal or risk of bias assessment is not typically required in scoping reviews, though subsequent refinements of the framework suggest that a quality appraisal may be considered in specific contexts such as when the goal is to inform clinical policy, or compare intervention effectiveness (though they highlight that formal tools, such as a risk of bias tool may not be useful if there is a broad variation in study designs included) [14,15]. Providing structured commentary on study quality can offer valuable context for interpreting findings and identifying directions for future research. Based on preliminary searches revealing substantial heterogeneity in study design, sample characteristics, and technological maturity, we determined that a formal risk of bias tool would not yield meaningful comparative insights. Instead, we included key indicators of methodological quality, including sample size, population features, use of comparators, randomization, outcome measurement, and tool validation in our data extraction table. This data was used to inform a structured narrative appraisal of strengths and limitations across the included studies.

## Results

### The Scope of Recent Evidence for Evaluating Digital Innovations in ABI Assessment

**Figure 1:**
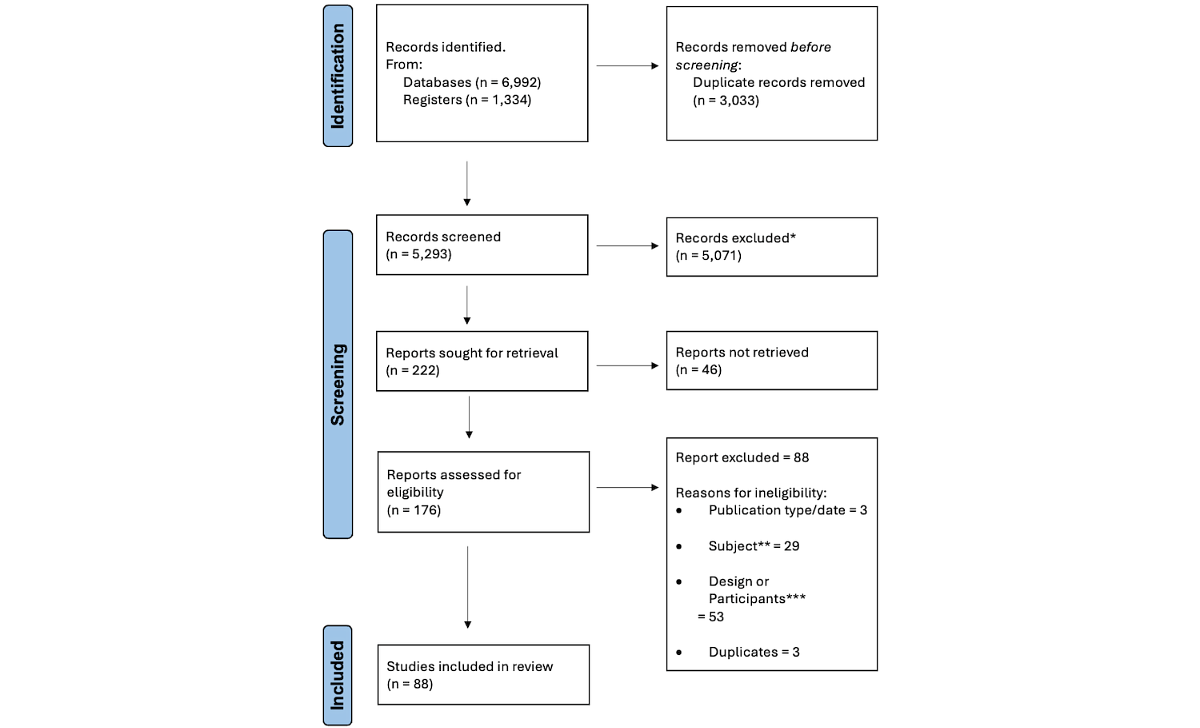
PRISMA-ScR (Preferred Reporting Items for Systematic Reviews and Meta-Analyses extension for Scoping Reviews) flowchart outlining the results from the review and selection process. * Excluded following title and abstract screening. **Excluded due to subject not fitting review criteria, that is, studies that do not evaluate the use of digital technology for clinical assessment. ***Excluded due to design or participants not fitting review criteria, that is, studies which do not include human participants with acquired brain injury, or review studies.

Figure 1 represents a PRISMA-ScR flowchart describing the results from the review and selection process. The search revealed 5293 unique articles, with 88 found to be relevant following the screening process**.**

### Technology Platforms and Apps

Most studies (n=45) focus on the use of smartphone (n=37) or tablet computer (n=8) based platforms for assessment of individuals with ABI. Most studies specified that they used a proprietary or commercial app to carry out assessments. Twenty-three studies describe computer-based (n=11) or web-based (n=12) tools; 11 studies used a telemedicine or teleconferencing platform; 9 studies used a VR platform, which included computer and VR eyewear. Five studies described using machine learning or artificial intelligence techniques alongside either a smartphone, tablet computer, computer, or web-based platform.

### How Technology Platform Use Changed Over Time

**Figure 2:**
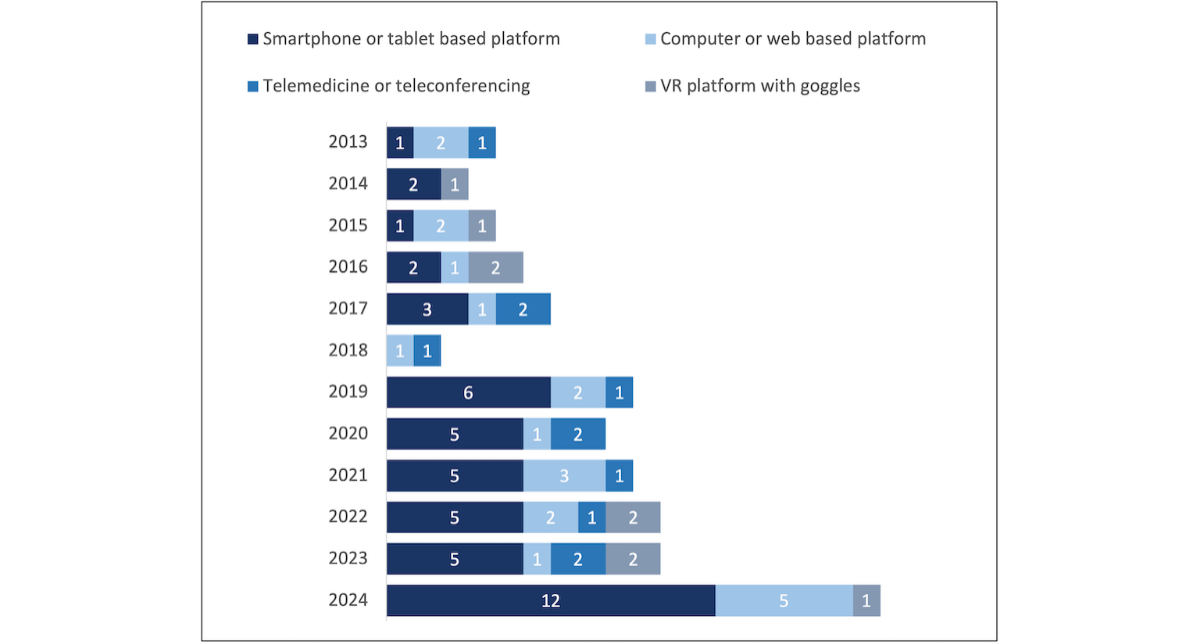
Trends in technology platforms studied over time. VR: virtual reality.

Figure 2 presents the main technology platforms used from 2013 to 2024, with an increasing proportion of studies using smartphone or tablet-based tools over time.

### Participant Characteristics and Setting

The median number of participants with ABI in each study was 25. There were 47 studies that included participants without an ABI; the median number of control participants in these studies was 50. Most studies included adult or late adolescent-adult participants and were not in a specific demographic group beyond their geographic area. Table 1 summarizes the site, ABI cohort sizes, and setting of the included studies, as well as the number of studies that focused on specific subpopulations (ie, pediatric, athletic, or military). Regarding the etiologies for participants with ABI, 79.5% (n=70) of studies included TBI as the sole etiology, 18.2% (n=16) included a variety of etiologies including with TBI, encephalitis, ischemic stroke, hemorrhagic stroke, neoplasm, epilepsy or were unspecified; 2 studies included a single etiology, namely neoplastic disease [29] and focal epilepsy [30]. A non-ABI cohort was included in 53.4% (n=47) of studies. This included 40 studies with healthy controls, 3 studies with participants with other morbidities (including orthopedic injury, pain, psychosis, or a history of trauma), and 4 studies that included both healthy and comorbid participants alongside participants with ABI.

**Table 1:** Setting and population characteristics.

		Number of studies (n=88), n	References
**Sites**			
	Single site study	71	[29,31-101]
	Multisite study	5	[102-106]
	Nationwide	9	[107-115]
	Multinational	3	[30,116,117]
**ABI^a^ cohort population size**			
	<20	29	[29,33,35,37,41,45,47,49,57,58,60,62,64,65,68,76,78,81,85-91,105,111,112,115]
	20-40	27	[32,34,40,42,43,48,50,52,55,56,63,67,69,72,75,76,79,80,82-84,92,93,95,97,100,117]
	41-100	18	[36,44,46,51,59,66,70,73,74,98,99,103,104,106,108,110,113,116]
	101-200	13	[31,38,53,54,61,71,94,96,101,107,109,114]
	>200^b^	2	[30,102]
**Specific populations**			
	Pediatric	5	[42,78,79,95,97]
	Athletes/sports	11	[32,43,49,51,60,62,80,81,83,113,114]
	Military (soldiers or veterans)	7	[38,46,72,85,98,104,105]
**Setting**			
	Laboratory or unspecified	24	[45,47,49,54,55,60,64,65,67,71,72,74,82-90,101,104,114]
	Community	37	[29-31,34-38,40,41,43,44,46,58,59,62,68-70,76,81,94,98-100,103,105-113,115,116]
	Emergency department	5	[56,57,73,75,95]
	Inpatient	6	[33,52,61,63,66,91]
	Outpatient clinic	16	[32,42,48,50,51,53,76,78-80,92,93,96,97,102,117]

^a^ABI: acquired brain injury.

^b^408 and 4999 participants, respectively.

### Themes of ABI Assessment

Following data charting, the study authors identified 5 overarching themes, based on the type of clinical assessment that was being studied. These themes were as follows:

1. Screening for a diagnosis of TBI, either acute or historical.
2. Monitoring or evaluating common subjective symptoms of TBI, for example, headache symptoms, sleep issues, psychological symptoms, or a combination.
3. Objective assessment of cognition—either cognitive screening or more detailed cognition or memory testing.
4. Objective assessment of language or communication.
5. Clinical consultation—using technology as an aid to the entire process of assessment and treatment for an individual with ABI.

The following is a narrative synthesis of the included studies under each theme, including a description and comparison of the technology platforms used and specific apps leveraged for assessment, study design, population characteristics, outcome measures, and main findings. This synthesis provides an overview of the current quality of evidence and validity of findings. Multimedia Appendix 3 [29-38,40-107,109-118] is a data table organizing studies by theme, technology platform, and specific app, providing a comprehensive synthesis of study design, population characteristics, outcome measures, and main findings

### TBI Detection or Screening

Fifteen studies focused on aiding the screening or diagnosis of a mild traumatic brain injury (mTBI) acutely [41,56,57,62,72,81,82,106,114,115], or screening for a distant history of TBI [60,103,108,110,116].

Several novel techniques to aid acute mTBI screening were demonstrated, especially the analysis of voice and video captured parameters. Yadav et al [106] carried out a large cohort study, recruiting from 47 high schools in the United States; they evaluated several machine learning techniques to analyze speech patterns in 95 participants at baseline and after mTBI, as well as a larger control cohort (n=486) and found the analysis software could be trained to be “reasonably” predictive of SRC (area under the curve=0.7). While the findings are promising, there was a lack of information on participant demographics, symptoms, and recovery, and we did not find further studies to validate these findings or address the real-world implementation of this tool [106]. The potential benefit of this approach is suggested by smaller studies of similar speech analysis tools, as well as studies combining speech and video analysis, and passive sensors of smartphone use [41,62,74,82,115]. However, the quality of evidence remains limited as the study methodology and outcome measures varied widely. There is also little evidence on the implementation of these tools in a larger setting, alongside more established screening protocols or compared with surveys on symptom burden.

We identified 4 studies which evaluate adaptations of the Ohio State University Traumatic Brain Injury Identification Method designed to screen for a lifetime history of TBI. These adaptations, including web-based surveys, computer-assisted telephone interviews, consistently demonstrated feasibility and good test-retest reliability for assessing lifetime TBI history across diverse cohorts; however, there was some variation in how this tool was adapted for digital use, the populations studied, and outcome measures, somewhat limiting the validity of findings [103,108,110,116].

### Symptom Assessment and Monitoring

Twenty-seven studies leveraged technology platforms to screen for, monitor, or quantify subjective symptoms related to TBI [29,31,33-38,40-44,58,68-70,94,96,98-100,105,107,109,111,112].

Many studies leveraged digital tools to monitor post-ABI symptoms and patient-reported outcomes [69,70,94,107,109]. We found several investigations on the feasibility of web-based, or computer-adapted telephone and text-message-based surveys. Survey completion rate was mixed and tended to reduce over time. Karvandi et al [107] provided serial surveys to 200 mTBI survivors and found <50% completion of all 3; however, importantly, the vast majority of respondents found the surveys to be useful. This was echoed by a small randomized trial by Suffoletto et al [70], using daily text-message questionnaires as well as tips on self-management, and found no significant improvement in mood or anxiety outcomes but reported high patient satisfaction with the program. This may suggest that though engagement may be challenging, patients may perceive value in these methods of monitoring.

Ecological momentary assessment (EMA)—the real-time logging of symptoms and experiences in a patient’s natural environment [119]—was another area of extensive research. Two pilot RCTs of EMA-based interventions in TBI showed no significant differences in clinical outcomes compared with standard care, but did demonstrate high user engagement and satisfaction rates, similar to trials on survey implementation described above [31,38]. Our review found many small cohort studies investigating the feasibility of smartphone-based EMA apps prompting patients with ABI to report on sleep quality, pain, mood, or activity levels multiple times a day [31,37,98,111,112] some of these investigations paired the EMA surveys with wearable sensors (such sleep and heart rate monitors), rate of compliance varied widely though it tended to be well regarded by participants. Several studies identified barriers to access, including cognitive impairments, health literacy, and memory deficits [31,34,99]. Sherer et al [31] outline several approaches to mitigate some of these barriers through regular phone calls to encourage engagement, and achieved a mean ≈80% response rate to EMA prompts. Overall, EMA was generally well tolerated, with many participants reporting it as useful, but there is a lack of evidence of its accuracy and impact on clinical outcomes across larger cohorts or longer periods.

### Physical Examination

We identified 16 studies that leveraged digital tools to assist in the physical examination of individuals with complications of ABI [45-52,73,75,83-85,89,91,92,102,104,113], including the evaluation of gait, balance, motor power, vision, and visuospatial function. We identified nine studies that used smartphone or VR sensor data (accelerometers and gyroscopes) to evaluate balance, gait, or posture in patients with TBI, though most studies were in the pilot phase [46-49,83,85,104,113]—the only products evaluated in more than one study were Sway Balance (Sway Medical) and AccWalker (University of North Carolina). AccWalker was investigated in a cross-sectional study and found to be sensitive in identifying neuromotor changes postblast exposure in soldiers. In a later study, AccWalker was further evaluated among a cohort of soldiers and civilians exposed to mTBI versus controls. The vast array of sensor information was leveraged to identify a highly sensitive parameter for identifying concussion (variability of max velocity), evaluated among a cohort of 62 participants with mTBI and 154 healthy controls [46,104]. Sway Balance was also found to differentiate between athletes with mTBI and balance impairment versus controls [48,50]. The performance of these smartphone-based products has not yet been adequately validated, however, as the methodology, participant characteristics, and outcome measures of all studies were widely different. There remains a lack of validation among larger, more representative groups of TBI patients across multiple sites, nor efforts to replicate findings of pilot studies with standardized outcome measures.

Another area of interest is using smartphone technology to perform neurological pupil examinations. Four studies examined smartphone-based measurement of the pupillary light reflex (PLR) for TBI assessment. One large retrospective review (which included ≈28,000 ophthalmology outpatients) on the use of the BrightLamp app (Brightlamp, Inc) and reported a strong predictive value of a specific composite of PLR measures for mTBI, controlling for age and gender, though the assessors were not blinded to the diagnosis [102]. We identified reports of the smartphone pupillometry app PupilScreen (UbiComp Lab, University of Washington) studied prospectively in two settings (inpatient and ophthalmology OPD) [52,91], while another app, ReflexPro (Brightlamp, Inc), was also studied in laboratory setting, all consistently reported to be sensitive to PLR changes in TBI controls [51], though they varied in terms of outcome measures and study design. These findings are promising, but further trials are needed to confirm the clinical impact of digital PLR tools, including in moderate-to-severe ABI, where they might serve as low-cost alternatives to specialized equipment in neurocritical care settings, especially in under-resourced regions [120]. There is limited but growing evidence that smartphone-based pupillometry may be comparable to dedicated pupillometry devices for a variety of ophthalmologic conditions, such as glaucoma [113], as well as for monitoring sedation in the neurocritical care settings [120,121], though these applications also lack validation or rigorous meta-analysis.

Other areas of interest were in the evaluation of telemedicine facilitated clinical examination [75,92], as well as machine learning assisted video-based neurological examination of limb movement [73]. While promising, study cohorts were small, and we did not identify follow-up research to validate the findings or investigate their clinical application and implementation.

### Cognitive Assessment

A variety of smartphone, computer-based, and VR tools were evaluated in 20 studies to help assess cognitive function in ABI [30,45,53-55,61,63-67,71,83,86-89,95,101].

A number of relatively large studies demonstrated promising findings: Pellinen et al [30] enrolled 408 individuals worldwide who were diagnosed with focal epilepsy to explore factors which impact completion of a web-based cognitive battery—they found male participants, and native English speakers to be more likely to engage whereas Black participants and participants with learning disabilities were less likely to engage—their work highlights barriers to receiving adequate clinical assessment and important areas to address as research moves towards implementation. The previously validated IMPACT screen for cognitive post TBI was adapted for tablet computer use [61] as well as multiplatform web-based use [114] and evaluated among 118-179 individuals with ABI and similar numbers age/gender matched of controls, both studies were sensitive in identifying cognitive changes in mTBI, though both were limited by narrow patient cohorts (trauma survivors and athletes respectively). Another tool which saw validation across 2 studies was the digital neuropsychological assessment, a web-based cognitive battery studied in TBI and control cohorts, with acceptable participant engagement, and significant cognitive differences between cohorts; however in a separate arm of one study, 30% of healthy participants scored in the 10th percentile for paper based tests, suggesting the reliability of digitally adapted assessments may be limited and requires careful evaluation [54,101]. Several smaller studies also evaluated adapted versions of cognitive assessments or a battery of tests for digital use; with most demonstrating feasibility and correlation with in-person or paper-based assessments, though with limited analysis of reliability of findings [53,56,71,95,101].

We identified reports on the development and evaluation of a digital task or tasks in a virtual [63,65,66,87,88,90] or augmented reality [45] environment, designed to test a range of cognitive domains and compared with healthy controls or benchmark paper-based tests. The largest “task-based” assessment included was described by Nadler et al [66], who developed the “internet bill paying task,” a high-fidelity web-based task that accurately differentiated between mTBI and healthy controls in a nonrandomized cross-sectional study, it also correlated well with standardized measures of executive dysfunction and impaired verbal fluency, however this study and other task based assessments while promising, were not found to be validated beyond the findings of a single study and a single site and did not address potential barriers such as lack of digital skills or injury-related vision changes which may lead to spurious results.

### Language or Communication Assessment

Four studies were identified evaluating the use of telemedicine to aid in assessment of ABI-related language or communication assessments [76,77,93,117]; this does not include several studies that assessed technology-based analysis of speech parameters to screen for acute mTBI, as discussed above. One small, randomized crossover trial, and one nonrandomized controlled study found that therapist-led evaluations of communication, using standardized tools over telemedicine platforms, yielded results comparable to in-person assessments carried out by separate assessors on the same individuals [76,77]. Telemedicine was found to be feasible and potentially effective for standardized assessment of discourse quality [93,117], for example, a trial by Turkstra et al [93], identified strong concordance between in-person and telemedicine-based assessment of participant conversation, but highlighted that this was limited by small sample size (n=20) and that visual or cognitive issues may pose a barrier to engaging with telehealth assessments at a wider level. Like other assessment domains, the scope of evidence remains limited and disparate, with a need for further validation and studies on wider implementation.

### Comprehensive Consultation

Three studies examined telehealth consultations for ABI patients in remote or underserved settings [78,79,97] and while on study investigated the use of telehealth consultation to provide a rapid consultation service post mTBI for athletes [80]. The included studies were pilot, feasibility, and qualitative in design, with outcome measures centered on user-satisfaction, cost-effectiveness or feasibility; the efficacy of these technologies have yet to be evaluated in larger populations or across multiple sites. Several studies did indicate high levels of patient satisfaction with telehealth services, and significant estimated savings on travel costs [80,97]. Despite this, telehealth platforms often rely heavily on internet connectivity and patient familiarity with digital technology, none of the included studies evaluate the impact of potential barriers to access, such as individuals with limited digital literacy, or those whose symptoms may be exacerbated by screen use; nor do they address how these potential barriers may lead to a sampling bias [122].

## Discussion

### Principal Findings

This scoping review identified a growing and diverse array of digital tools being used to screen, assess, and monitor complications of ABI across different populations and clinical settings. Advances in mobile apps, web platforms, telehealth, machine learning, and VR have enabled innovative approaches, with the most prominent themes found in the screening/identification of TBI, symptom assessment, physical examination, cognition and language assessment, as well as facilitation of a general consultation. While some digital assessment apps show promising preliminary evidence, many areas remain understudied, and the tools included lacked standardization across studies. The goal of this scoping review was to provide a comprehensive overview and synthesis of recent research, which included details on study characteristics without a formal assessment of quality; therefore, it provides limited insight into the efficacy of specific platforms or tools, or how they may serve a wider population. For all the themes identified, future work should focus on validation of findings, approach questions of implementation and scalability in real-world settings, and address ethical concerns such as barriers to access and data privacy.

### Gaps and Limitations in Current Evidence

Although this review mapped a broad range of digital innovations, the overall evidence base has significant limitations. The studies were highly heterogeneous in terms of participant populations, technology types, and outcome measures. Most involved small, or specific cohorts (eg, exclusively athletes or military personnel), which limits generalizability. Few technologies were evaluated in more than one study or site. Notable exceptions involved the Ohio State University Traumatic Brain Injury Identification Method survey and several balance assessment apps, each examined in at least two studies; however, methods and outcome measures varied between those studies. As a result, most digital tools identified in this review lack robust validation across diverse clinical settings. Rigorous study designs were also scarce: we found five randomized controlled trials in total (all of them small feasibility studies), while the majority of studies were observational and many lacked control groups. These weaknesses mean the effectiveness and clinical utility of many digital assessment tools remain unconfirmed. This issue is not unique to ABI, with similar limitations identified in other scoping reviews of digital health across domains of depression, cancer rehabilitation, and aging [16-18].

### Barriers to Implementation and Stakeholder Engagement

The development and implementation of digital tools also remains ad-hoc, with almost no reference to implementation strategy or the involvement of stakeholders in co-design, key features to maximize the utility and acceptance of an innovative tool [123]. There has been growing acceptance of application theory across other areas of ABI research, including in education campaigns and new rehabilitation strategies [124]. These frameworks should help guide the wider introduction of potentially useful tools.

### Ethical, Accessibility, and Data Security Considerations

Discussion on potential risks, ethical concerns, and data security was limited. Several studies explicitly addressed local data protection law compliance [72,86], or the use of an encrypted server or database [31,35,36,41,116], though the majority did not present detailed information on the exact data privacy and security protocols. Another area that is important to address is accessibility, while technology does have the potential to improve access, potential barriers to access, such as visual/hearing impairments, poor health literacy, or lack of internet access, were rarely addressed or actively mitigated [122]. Most developed countries have detailed data privacy and security laws, such as the Health Insurance Portability and Accountability Act and General Data Protection Regulation in the United States and the European Union, respectively [125]. It is vital that strict adherence to these laws is demonstrated as a minimum standard as research continues to grow. International bodies such as the WHO have developed guidelines on the safe integration and evaluation of digital health technologies across health services [23]. These can serve as a framework for safe, ethical, and just implementation strategies for promising technologies in the future.

### Priorities for Future Research

Larger, more rigorous studies (ideally across multiple centers and in real-world clinical settings) are needed to confirm the reliability and effectiveness of these digital tools. Validation is especially important when the digital tool is involved in making a “diagnosis” such as in mTBI screening or cognitive assessment—though many studies showed promise in aiding these diagnoses, inaccurate findings could lead to misdiagnosis and significant harms; a key focus should be on ensuring a standardized approach to evaluating digital tools: with studies that include similar cohorts, experimental designs and outcome measures. More evidence is required on how access to innovative tools may impact overall recovery; this will again require studies with much larger cohorts and longer follow-up times. We need to better understand how to implement these tools across the scope of care and ensure adequate penetrance when a tool is effective, adherence to implementation strategies [114], not just identifying barriers to access but actively mitigating them [60,62]. More work is also needed to provide better assessment and monitoring in all cases of ABI (not just mTBI or specific populations such as athletes), and for complications such as seizures, behavioral symptoms, and participation restrictions.

### Conclusions

This review highlights major strides in the application of technology to help improve how individuals with ABI are screened and assessed. There is a particular focus on mTBI, with less published on assessments of patients with moderate to severe TBI, or other aetiologies of ABI. Most studies performed to date include small numbers of patients, designed as pilot or feasibility studies; thus, more rigorous research is needed to better understand the efficacy and applicability of various technologies. Future developments should also consider the assessment of less-explored complications of ABI, and leverage assessments across multiple domains to provide holistic care. It is important to ensure that this work considers the ethical implications of the technologies involved, including issues such as accessibility, digital literacy, privacy, and security.

Though this synthesis can help guide research in promising areas, or where there are gaps, more steps are needed to help guide the implementation of a specific innovation or policy, such as a systematic review with a more narrow focus and a formal risk of bias tool.

This review should inform the development of further digital tools to provide a comprehensive and equitable approach to clinical assessment in patients with ABI, which is accessible and efficacious for all.

## Appendix

Multimedia Appendix 1 PRISMA-ScR checklist.

Multimedia Appendix 2 Search strategy, search string, reference definitions and inclusion/exclusion criteria.

Multimedia Appendix 3 Supplementary Table outlining the data extracted from all included reports.

## Abbreviations

ABI: acquired brain injury
EMA: ecological momentary assessment
MeSH: Medical Subject Headings
mTBI: mild traumatic brain injury
PLR: pupillary light reflex
PRISMA-ScR: Preferred Reporting Items for Systematic Reviews and Meta-Analyses extension for Scoping Reviews
TBI: traumatic brain injury
VR: virtual reality
