# Supplementary material for: Digital Innovations for Clinical Assessment in Acquired Brain Injury: Scoping Review
Source: J Med Internet Res. 2025 Nov 5;27:e73331. doi: 10.2196/73331 (PMC12631094; doi:10.2196/73331)
Supplement: Multimedia Appendix 2 [file jmir_v27i1e73331_app2.docx]

Search and Screening Strategy

**Title:** Digital Innovations for Clinical Assessment in Acquired Brain Injury: Scoping Review

**Aim**: To identify and map research on what literature exists describing the development or implementation of ehealth, online or mobile app-based tools aimed to assist medical professionals in the screening and assessment of acquired brain injury and its complications in the acute, rehabilitation and community setting.

# PICO Format

**Patient**: Individuals with acquired brain injury

**Intervention**: Digital tools that assist with screening, diagnosis, assessment and monitoring of complications of brain injury.

**Comparison**: NA

**Outcome**: Mapping and synthesis of current evidence

# Developing the Search Strategy

Prior to developing a search string, definitions for the key concepts of the study were agreed upon. ABI was defined as any individual who suffered an injury to the brain that was not developmental in nature or acquired at birth [1], due the unique presentation and specific needs of individuals with stroke [2], it was agreed not to include this cohort in the scoping review. Digital health technologies were defined in accordance with the U.S. Food and Drug Administration (FDA) as encompassing mobile health (mHealth), health IT, wearable devices, telemedicine, and AI-assisted tools for healthcare assessment and monitoring [3]. Assessments were defined as any method to screen for, evaluate, quantify or monitor ABI or its complications. The context was defined as any digital technology being used to aid assessment, monitoring or screening tests on individuals with ABI in any health setting.

The search was applied across MEDLINE (via Ovid), EMBASE (via Ovid), and Scopus. A comprehensive search string was developed by combining controlled vocabulary (e.g., MeSH terms) and free-text terms related to three core concepts: (1) ABI (e.g., “acquired brain injury”, “traumatic brain injury”, “brain tumour”, “encephalitis”, ), (2) digital technologies (e.g., “telemedicine”, “mobile health”, “eHealth”, “apps”, “wearables”, “artificial intelligence”), and (3) clinical processes (e.g., “assessment”, “screening”, “monitoring”, “measurement”). Boolean operators (“AND”, “OR”) and truncation were used to combine synonyms and refine results. Each database-specific syntax was adapted to its indexing system. The development process included iterative testing and refinement of the search terms and the review of reference lists of included studies to identify additional keywords. The

A strategy for including Gray Literature was also confirmed A Priori. Scopus was used for its innovative feature that searches across multiple pre-print databases, Embase’s feature for screening for conference abstracts was implemented, which were then used to search for full text articles both published and/or preprints. As well as research databases , 4 clinical trials registries were searched using broad search terms: the International clinical trials registry, clinicaltrials.gov, the EU Clinical trials register and the UK’s ISTRCN (originally called International Standard Randomized Controlled Trial Number).

# Search String

## Medline (PUBMED) Search

| **#** | **Query** | **Results from 16 Feb 2025** |
| --- | --- | --- |
| 1 | Mobile Applications/ | 12,209 |
| 2 | exp Internet/ | 99,633 |
| 3 | exp Cell Phone/ | 23,275 |
| 4 | exp Computers, Handheld/ | 13,651 |
| 5 | Medical Informatics Applications/ | 2,552 |
| 6 | Therapy, Computer-Assisted/ | 6,979 |
| 7 | (app or apps).ti,ab. | 46,067 |
| 8 | (online or web or internet or digital*).ti. | 146,305 |
| 9 | ((online or web or internet or digital*) adj3 (based or application* or intervention* or program* or therap*)).ab. | 84,221 |
| 10 | (phone* or telephone* or smartphone* or cellphone* or smartwatch*).ti. | 28,325 |
| 11 | ((phone* or telephone* or smartphone* or cellphone* or smartwatch*) adj3 (based or application* or intervention* or program* or therap*)).ab. | 17,994 |
| 12 | (mobile health or mhealth or m-health or ehealth or e-health or emental or e-mental).ti. | 9,075 |
| 13 | ((mobile health or mhealth or m-health or ehealth or e-health or emental or e-mental) adj3 (based or application* or intervention* or program* or therap*)).ab. | 6,349 |
| 14 | (mobile* adj3 (based or application* or intervention* or device* or technolog*)).ti,ab. | 23,341 |
| 15 | or/1-14 | 360,518 |
| 16 | exp Brain Injuries, Traumatic/ or exp Brain Injuries/ | 84,389 |
| 17 | exp Brain Injuries, Diffuse/ | 873 |
| 18 | exp Brain Injury, Chronic/ | 1,200 |
| 19 | exp Craniocerebral Trauma/ | 181,829 |
| 20 | ((brain or head or cerebral) adj3 (injury or injuries or trauma)).ab,ti. | 130,090 |
| 21 | exp Hypoxia, Brain/ | 14,517 |
| 22 | exp Hypoxia-Ischemia, Brain/ | 7,178 |
| 23 | ((brain or head or cerebral) adj3 (anoxic or anoxia or hypoxia)).ab,ti. | 6,180 |
| 24 | "Encephalopath*".ab,ti. | 60,334 |
| 25 | exp Coma, Post-Head Injury/ | 128 |
| 26 | exp Brain Concussion/ | 12,739 |
| 27 | (concussion* or Concussive or Neurotrauma*).ab,ti. | 14,619 |
| 28 | exp Sepsis-Associated Encephalopathy/ | 295 |
| 29 | exp Hypertensive Encephalopathy/ | 2,254 |
| 30 | exp Wernicke Encephalopathy/ | 1,921 |
| 31 | exp Acute Febrile Encephalopathy/ | 99 |
| 32 | Chronic Traumatic Encephalopathy/ | 419 |
| 33 | 16 or 17 or 18 or 19 or 20 or 21 or 22 or 23 or 24 or 25 or 26 or 27 or 28 or 29 or 30 or 31 or 32 | 313,852 |
| 34 | exp Symptom Assessment/ | 7,109 |
| 35 | (screen* or evaluat* or monitor* or assess* or identif* or complication*).ab,ti. | 11,780,274 |
| 36 | (apprais* or detect* or interpret* or diagnos* or recogni* or attribut* or intervention*).ab,ti. | 7,995,993 |
| 37 | 34 or 35 | 11,782,562 |
| 38 | 34 or 35 or 36 | 15,612,967 |
| 39 | 15 and 33 and 37 | 1,368 |
| 40 | 15 and 33 and 38 | 1,663 |
| 41 | exp Telemedicine/ | 46,643 |
| 42 | exp Remote Consultation/ | 5,889 |
| 43 | exp Telecommunications/ | 127,610 |
| 44 | exp Videoconferencing/ | 2,774 |
| 45 | exp Hotlines/ | 2,967 |
| 46 | ((remote or virtual or digital) adj3 (evaluation* or consult* or management or assessment or clinic* or intervention)).ab,ti. | 16,548 |
| 47 | ("Tele Referral" or "Tele-Referral" or telereferral).ab,ti. | 6 |
| 48 | (telemedic* or tele-medic* or tele medic*).ab,ti. | 20,481 |
| 49 | (Tele-assistance or Tele assistance or teleassistance).ab,ti. | 83 |
| 50 | "Virtual Medicine".ab,ti. | 47 |
| 51 | (telehealth or tele-health or "tele health").ab,ti. | 12,314 |
| 52 | (Telecare or Tele-Care or "Tele Care").ab,ti. | 839 |
| 53 | (tele-rehab* or tele rehab* or telerehab*).ab,ti. | 2,063 |
| 54 | (telediagnos* or tele-diagnos* or "tele diagnos*").ab,ti. | 279 |
| 55 | (telemonitor* or tele-monitor* or "tele monitor").ab,ti. | 2,500 |
| 56 | (e-consultation* or "e consultation*" or econsultation*).ab,ti. | 218 |
| 57 | (Teleconsultation* or "tele consultation*" or tele-consultation*).ab,ti. | 2,200 |
| 58 | "Telecommunication*".ab,ti. | 5,584 |
| 59 | "Teleconferenc*".ab,ti. | 1,567 |
| 60 | (Videoconferenc* or "Video Conferenc*").ab,ti. | 5,712 |
| 61 | "Hotline*".ab,ti. | 1,608 |
| 62 | 41 or 42 or 43 or 44 or 45 or 46 or 47 or 48 or 49 or 50 or 51 or 52 or 53 or 54 or 55 or 56 or 57 or 58 or 59 or 60 or 61 | 164,743 |
| 63 | 15 or 62 | 468,061 |
| 64 | 33 and 37 and 63 | 1,832 |
| 65 | 33 and 38 and 63 | 2,192 |
| 66 | 65 and 2012:2024.(sa_year). | 1,582 |
| 67 | 39 and 2012:2024.(sa_year). | 1,018 |
| 68 | 40 and 2012:2024.(sa_year). | 1,211 |
| 69 | 64 and 2012:2024.(sa_year). | 1,353 |
| 70 | 66 not 69 | 229 |
| 71 | 66 not 68 | 371 |
| 72 | 66 not 67 | 564 |

**Link:**
[**Click to run search**](https://ovidsp.ovid.com/athens/ovidweb.cgi?T=JS&NEWS=N&PAGE=main&SHAREDSEARCHID=32vzkevDArtHT8yStkuanZhp5AygJAnr8XCR85Af5e33HCgYB0J7iRjZb240AvczW)

## Embase Search

| **Search Sequence** | **Searches** | **Results as of Feb 23^rd 2025^** |
| --- | --- | --- |
| 1. 1 | exp "mobile application"/ | 27437 |
| 1. 2 | exp internet/ | 132663 |
| 3 | exp mobile phone/ | 50295 |
| 4 | text messaging/ | 8111 |
| 5 | personal digital assistant/ | 1862 |
| 6 | computer assisted therapy/ | 4873 |
| 7 | (app or apps).ti,ab. | 62512 |
| 8 | (online or web or internet or digital*).ti. | 167021 |
| 9 | ((online or web or internet or digital*) adj3 (based or application* or intervention* or program* or therap*)).ab. | 112194 |
| 10 | (phone* or telephone* or smartphone* or cellphone* or smartwatch*).ti. | 33443 |
| 11 | ((phone* or telephone* or smartphone* or cellphone* or smartwatch*) adj3 (based or application* or intervention* or program* or therap*)).ab. | 23845 |
| 12 | (mobile health or mhealth or m-health or ehealth or e-health or emental or e-mental).ti. | 9912 |
| 13 | ((mobile health or mhealth or m-health or ehealth or e-health or emental or e- mental) adj3 (based or application* or intervention* or program* or therap*)).ab. | 6919 |
| 14 | (mobile* adj3 (based or application* or intervention* or device* or technolog*)).ti,ab. | 28532 |
| 15 | exp "telecommunication"/ | 122447 |
| 16 | (telemed* or Telehealth or tele-health Telecommunication or tele-care or teleassistance or tele-assistance or telediagnosis or telediagnosis or telemonitoring or tele-monitoring or telerehab* or Tele-rehab or Teleconferenc* or tele-conferenc* or vidoconferenc* or video-conferenc* or teleconsultation or tele-consultation or e-consultation or econsultation or hotline*).ti,ab. | 54352 |
| 17 | (Virtual and Med*).ti. | 2163 |
| 18 | (remote and Consult*).ti. | 413 |
| 19 | (tele and Care).ti. | 459 |
| 20 | (tele and assist*).ti. | 71 |
| 21 | (tele and diagnosis).ti. | 53 |
| 22 | (tele and Monitoring).ti. | 137 |
| 23 | (video and conferenc*).ti. | 273 |
| 24 | (tele and Rehab*).ti. | 249 |
| 25 | (tele and Consult*).ti. | 108 |
| 26 | (tele and conferenc*).ti. | 16 |
| 27 | or/1-26 | 563775 |
| 28 | exp "Brain Injury"/ | 219312 |
| 29 | ((brain or head or cerebral) adj3 (injury or injuries or trauma)).ab,ti. | 177235 |
| 30 | (neurotrauma or "anoxic brain" or "encephalop*" or "concuss*" or "post-concussion*" or "mTBI" or "TBI" or "SRC" or ABI).ab,ti. | 222327 |
| 31 | 28 or 29 or 30 | 446113 |
| 32 | exp "assessment of humans"/ | 2032263 |
| 33 | (screen* or evaluat* or monitor* or assess* or identif* or complication*).ab,ti. | 15895491 |
| 34 | 32 or 33 | 16512134 |
| 35 | 27 and 31 and 34 | 3307 |
| 36 | 35 and 2013:2024.(sa_year). | 2509 |
|  | https://ovidsp.ovid.com/ovidweb.cgi?T=JS&NEWS=N&PAGE=main&SHAREDSEARCHID=2AIuo3iQwbKQ5yWragLWAckFOagiIrA5GUlqR83G98LPTMjUufc3IY3kJStzDEsrH |  |

## Scopus Search

| **Search Sequence** | **Searches** | **Results From Feb 28^th^ 2025** |
| --- | --- | --- |
| 1 | TITLE-ABS-KEY ( "mobile application*" ) | 62,237 results |
| 2 | TITLE-ABS-KEY ( "handheld computer*" ) | 1,461 results |
| 3 | TITLE-ABS-KEY ( "medical informatics application*" ) | 2,596 results |
| 4 | TITLE-ABS-KEY ( "computer assisted therapy" ) | 5,998 results |
| 5 | TITLE-ABS-KEY ( app OR apps ) | 115,675 results |
| 6 | TITLE ( online OR web OR internet OR digital* ) | 875,184 results |
| 7 | TITLE-ABS-KEY ( ( online OR web OR internet OR digital* ) W/2 ( based OR application* OR intervention* OR program* OR therap* ) ) | 409,191 results |
| 8 | TITLE ( phone* OR telephone* OR smartphone* OR cellphone* OR smartwatch* ) | 86,492 results |
| 9 | TITLE-ABS-KEY ( ( phone* OR telephone* OR smartphone* OR cellphone* OR smartwatch* ) W/2 ( based OR application* OR intervention* OR program* OR therap* ) ) | 50,010 results |
| 10 | TITLE ( "mobile health" OR mhealth OR m-health OR ehealth OR e-health OR emental OR e-mental ) | 16,105 results |
| 11 | TITLE-ABS-KEY ( ( "mobile health" OR mhealth OR m-health OR ehealth OR e-health OR emental OR e-mental ) W/2 ( based OR application* OR intervention* OR program* OR therap* ) ) | 11,393 results |
| 12 | TITLE-ABS-KEY ( mobile* W/2 ( based OR application* OR intervention* OR device* OR technolog* ) ) | 230,351 results |
| 13 | TITLE-ABS-KEY ( ( remote OR virtual OR digital ) W/2 ( evaluation* OR consult* OR management OR assessment OR clinic* OR intervention ) ) | 59,629 results |
| 14 | TITLE-ABS-KEY ( "tele referral" OR tele-referral OR telereferral ) | 17 results |
| 15 | TITLE-ABS-KEY ( telemedic* OR tele-medic* OR "tele medic*" ) | 73,645 results |
| 16 | TITLE-ABS-KEY ( tele-assistance OR "tele assistance" OR teleassistance ) | 297 results |
| 17 | TITLE-ABS-KEY ( "virtual medicine" ) | 113 results |
| 18 | TITLE-ABS-KEY ( telehealth OR tele-health OR "tele health" ) | 28,363 results |
| 19 | TITLE-ABS-KEY ( telecare OR tele-care OR "tele care" ) | 3,204 results |
| 20 | TITLE-ABS-KEY ( tele-rehab* OR "tele rehab*" OR telerehab* ) | 4,537 results |
| 21 | TITLE-ABS-KEY ( telediagnos* OR tele-diagnos* OR "tele diagnos*" ) | 1,072 results |
| 22 | TITLE-ABS-KEY ( telemonitor* OR tele-monitor* OR "tele monitor*" ) | 7,523 results |
| 23 | TITLE-ABS-KEY ( e-consultation* OR "e consultation*" OR econsultation* ) | 418 results |
| 24 | TITLE-ABS-KEY ( teleconsultation* OR "tele consultation*" OR tele-consultation* ) | 15,289 results |
| 25 | TITLE-ABS-KEY ( telecommunication* ) | 536,879 results |
| 26 | TITLE-ABS-KEY ( teleconferenc* ) | 8,399 results |
| 27 | TITLE-ABS-KEY ( videoconferenc* OR "video conferenc*" ) | 23,956 results |
| 28 | TITLE-ABS-KEY ( hotline* ) | 5,101 results |
| 29 | TITLE-ABS-KEY ( "remote consultation*" ) | 6,417 results |
| 30 | ( TITLE-ABS-KEY ( "mobile application*" ) ) OR ( TITLE-ABS-KEY ( "handheld computer*" ) ) OR ( TITLE-ABS-KEY ( "medical informatics application*" ) ) OR ( TITLE-ABS-KEY ( "computer assisted therapy" ) ) OR ( TITLE-ABS-KEY ( app OR apps ) ) OR ( TITLE ( online OR web OR internet OR digital* ) ) OR ( TITLE-ABS-KEY ( ( online OR web OR internet OR digital* ) W/2 ( based OR application* OR intervention* OR program* OR therap* ) ) ) OR ( TITLE ( phone* OR telephone* OR smartphone* OR cellphone* OR smartwatch* ) ) OR ( TITLE-ABS-KEY ( ( phone* OR telephone* OR smartphone* OR cellphone* OR smartwatch* ) W/2 ( based OR application* OR intervention* OR program* OR therap* ) ) ) OR ( TITLE ( "mobile health" OR mhealth OR m-health OR ehealth OR e-health OR emental OR e-mental ) ) OR ( TITLE-ABS-KEY ( ( "mobile health" OR mhealth OR m-health OR ehealth OR e-health OR emental OR e-mental ) W/2 ( based OR application* OR intervention* OR program* OR therap* ) ) ) OR ( TITLE-ABS-KEY ( mobile* W/2 ( based OR application* OR intervention* OR device* OR technolog* ) ) ) OR ( TITLE-ABS-KEY ( ( remote OR virtual OR digital ) W/2 ( evaluation* OR consult* OR management OR assessment OR clinic* OR intervention ) ) ) OR ( TITLE-ABS-KEY ( "tele referral" OR tele-referral OR telereferral ) ) OR ( TITLE-ABS-KEY ( telemedic* OR tele-medic* OR "tele medic*" ) ) OR ( TITLE-ABS-KEY ( tele-assistance OR "tele assistance" OR teleassistance ) ) OR ( TITLE-ABS-KEY ( "virtual medicine" ) ) OR ( TITLE-ABS-KEY ( telehealth OR tele-health OR "tele health" ) ) OR ( TITLE-ABS-KEY ( telecare OR tele-care OR "tele care" ) ) OR ( TITLE-ABS-KEY ( tele-rehab* OR "tele rehab*" OR telerehab* ) ) OR ( TITLE-ABS-KEY ( telediagnos* OR tele-diagnos* OR "tele diagnos*" ) ) OR ( TITLE-ABS-KEY ( telemonitor* OR tele-monitor* OR "tele monitor*" ) ) OR ( TITLE-ABS-KEY ( e-consultation* OR "e consultation*" OR econsultation* ) ) OR ( TITLE-ABS-KEY ( teleconsultation* OR "tele consultation*" OR tele-consultation* ) ) OR ( TITLE-ABS-KEY ( telecommunication* ) ) OR ( TITLE-ABS-KEY ( teleconferenc* ) ) OR ( TITLE-ABS-KEY ( videoconferenc* OR "video conferenc*" ) ) OR ( TITLE-ABS-KEY ( hotline* ) ) OR ( TITLE-ABS-KEY ( "remote consultation*" ) ) | 2,063,862 results |
| 31 | TITLE-ABS-KEY ( "traumatic brain injur*" ) | 75,504 results |
| 32 | TITLE-ABS-KEY ( "acquired brain injur*" ) | 4,550 results |
| 33 | TITLE-ABS-KEY ( "craniocerebral trauma" ) | 22,177 results |
| 34 | TITLE-ABS-KEY ( ( brain OR head OR cerebral ) W/2 ( injury OR injuries OR trauma ) ) | 258,879 results |
| 35 | TITLE-ABS-KEY ( ( brain OR head OR cerebral ) W/2 ( anoxic OR anoxia OR hypoxia ) ) | 24,519 results |
| 36 | TITLE-ABS-KEY ( encephalopath* ) | 100,417 results |
| 37 | TITLE-ABS-KEY ( concussion* OR concussive OR neurotrauma* ) | 26,348 results |
| 38 | TITLE-ABS-KEY ( coma* ) | 117,578 results |
| 39 | ( TITLE-ABS-KEY ( "traumatic brain injur*" ) ) OR ( TITLE-ABS-KEY ( "acquired brain injur*" ) ) OR ( TITLE-ABS-KEY ( "craniocerebral trauma" ) ) OR ( TITLE-ABS-KEY ( ( brain OR head OR cerebral ) W/2 ( injury OR injuries OR trauma ) ) ) OR ( TITLE-ABS-KEY ( ( brain OR head OR cerebral ) W/2 ( anoxic OR anoxia OR hypoxia ) ) ) OR ( TITLE-ABS-KEY ( encephalopath* ) ) OR ( TITLE-ABS-KEY ( concussion* OR concussive OR neurotrauma* ) ) OR ( TITLE-ABS-KEY ( coma* ) ) | 463,290 results |
| 40 | TITLE-ABS-KEY ( "symptom assess*" ) | 20,816 results |
| 41 | TITLE-ABS-KEY ( screen* OR evaluat* OR monitor* OR assess* OR identif* OR complication* ) | 26,112,846 results |
| 42 | TITLE-ABS-KEY ( apprais* OR detect* OR interpret* OR diagnos* OR recogni* OR attribut* OR intervention* ) | 18,005,063 results |
| 43 | TITLE-ABS-KEY ( "symptom assess*" ) ) OR ( TITLE-ABS-KEY ( screen* OR evaluat* OR monitor* OR assess* OR identif* OR complication* ) ) OR ( TITLE-ABS-KEY ( apprais* OR detect* OR interpret* OR diagnos* OR recogni* OR attribut* OR intervention* ) ) | 35,795,060 results |
| 44 | ( ( TITLE-ABS-KEY ( "symptom assess*" ) ) OR ( TITLE-ABS-KEY ( screen* OR evaluat* OR monitor* OR assess* OR identif* OR complication* ) ) OR ( TITLE-ABS-KEY ( apprais* OR detect* OR interpret* OR diagnos* OR recogni* OR attribut* OR intervention* ) ) ) AND ( ( TITLE-ABS-KEY ( "traumatic brain injur*" ) ) OR ( TITLE-ABS-KEY ( "acquired brain injur*" ) ) OR ( TITLE-ABS-KEY ( "craniocerebral trauma" ) ) OR ( TITLE-ABS-KEY ( ( brain OR head OR cerebral ) W/2 ( injury OR injuries OR trauma ) ) ) OR ( TITLE-ABS-KEY ( ( brain OR head OR cerebral ) W/2 ( anoxic OR anoxia OR hypoxia ) ) ) OR ( TITLE-ABS-KEY ( encephalopath* ) ) OR ( TITLE-ABS-KEY ( concussion* OR concussive OR neurotrauma* ) ) OR ( TITLE-ABS-KEY ( coma* ) ) ) AND ( ( TITLE-ABS-KEY ( "mobile application*" ) ) OR ( TITLE-ABS-KEY ( "handheld computer*" ) ) OR ( TITLE-ABS-KEY ( "medical informatics application*" ) ) OR ( TITLE-ABS-KEY ( "computer assisted therapy" ) ) OR ( TITLE-ABS-KEY ( app OR apps ) ) OR ( TITLE ( online OR web OR internet OR digital* ) ) OR ( TITLE-ABS-KEY ( ( online OR web OR internet OR digital* ) W/2 ( based OR application* OR intervention* OR program* OR therap* ) ) ) OR ( TITLE ( phone* OR telephone* OR smartphone* OR cellphone* OR smartwatch* ) ) OR ( TITLE-ABS-KEY ( ( phone* OR telephone* OR smartphone* OR cellphone* OR smartwatch* ) W/2 ( based OR application* OR intervention* OR program* OR therap* ) ) ) OR ( TITLE ( "mobile health" OR mhealth OR m-health OR ehealth OR e-health OR emental OR e-mental ) ) OR ( TITLE-ABS-KEY ( ( "mobile health" OR mhealth OR m-health OR ehealth OR e-health OR emental OR e-mental ) W/2 ( based OR application* OR intervention* OR program* OR therap* ) ) ) OR ( TITLE-ABS-KEY ( mobile* W/2 ( based OR application* OR intervention* OR device* OR technolog* ) ) ) OR ( TITLE-ABS-KEY ( ( remote OR virtual OR digital ) W/2 ( evaluation* OR consult* OR management OR assessment OR clinic* OR intervention ) ) ) OR ( TITLE-ABS-KEY ( "tele referral" OR tele-referral OR telereferral ) ) OR ( TITLE-ABS-KEY ( telemedic* OR tele-medic* OR "tele medic*" ) ) OR ( TITLE-ABS-KEY ( tele-assistance OR "tele assistance" OR teleassistance ) ) OR ( TITLE-ABS-KEY ( "virtual medicine" ) ) OR ( TITLE-ABS-KEY ( telehealth OR tele-health OR "tele health" ) ) OR ( TITLE-ABS-KEY ( telecare OR tele-care OR "tele care" ) ) OR ( TITLE-ABS-KEY ( tele-rehab* OR "tele rehab*" OR telerehab* ) ) OR ( TITLE-ABS-KEY ( telediagnos* OR tele-diagnos* OR "tele diagnos*" ) ) OR ( TITLE-ABS-KEY ( telemonitor* OR tele-monitor* OR "tele monitor*" ) ) OR ( TITLE-ABS-KEY ( e-consultation* OR "e consultation*" OR econsultation* ) ) OR ( TITLE-ABS-KEY ( teleconsultation* OR "tele consultation*" OR tele-consultation* ) ) OR ( TITLE-ABS-KEY ( telecommunication* ) ) OR ( TITLE-ABS-KEY ( teleconferenc* ) ) OR ( TITLE-ABS-KEY ( videoconferenc* OR "video conferenc*" ) ) OR ( TITLE-ABS-KEY ( hotline* ) ) OR ( TITLE-ABS-KEY ( "remote consultation*" ) ) ) | 3,265 results |
| 45 | ( ( TITLE-ABS-KEY ( "symptom assess*" ) ) OR ( TITLE-ABS-KEY ( screen* OR evaluat* OR monitor* OR assess* OR identif* OR complication* ) ) OR ( TITLE-ABS-KEY ( apprais* OR detect* OR interpret* OR diagnos* OR recogni* OR attribut* OR intervention* ) ) ) AND ( ( TITLE-ABS-KEY ( "traumatic brain injur*" ) ) OR ( TITLE-ABS-KEY ( "acquired brain injur*" ) ) OR ( TITLE-ABS-KEY ( "craniocerebral trauma" ) ) OR ( TITLE-ABS-KEY ( ( brain OR head OR cerebral ) W/2 ( injury OR injuries OR trauma ) ) ) OR ( TITLE-ABS-KEY ( ( brain OR head OR cerebral ) W/2 ( anoxic OR anoxia OR hypoxia ) ) ) OR ( TITLE-ABS-KEY ( encephalopath* ) ) OR ( TITLE-ABS-KEY ( concussion* OR concussive OR neurotrauma* ) ) OR ( TITLE-ABS-KEY ( coma* ) ) ) AND ( ( TITLE-ABS-KEY ( "mobile application*" ) ) OR ( TITLE-ABS-KEY ( "handheld computer*" ) ) OR ( TITLE-ABS-KEY ( "medical informatics application*" ) ) OR ( TITLE-ABS-KEY ( "computer assisted therapy" ) ) OR ( TITLE-ABS-KEY ( app OR apps ) ) OR ( TITLE ( online OR web OR internet OR digital* ) ) OR ( TITLE-ABS-KEY ( ( online OR web OR internet OR digital* ) W/2 ( based OR application* OR intervention* OR program* OR therap* ) ) ) OR ( TITLE ( phone* OR telephone* OR smartphone* OR cellphone* OR smartwatch* ) ) OR ( TITLE-ABS-KEY ( ( phone* OR telephone* OR smartphone* OR cellphone* OR smartwatch* ) W/2 ( based OR application* OR intervention* OR program* OR therap* ) ) ) OR ( TITLE ( "mobile health" OR mhealth OR m-health OR ehealth OR e-health OR emental OR e-mental ) ) OR ( TITLE-ABS-KEY ( ( "mobile health" OR mhealth OR m-health OR ehealth OR e-health OR emental OR e-mental ) W/2 ( based OR application* OR intervention* OR program* OR therap* ) ) ) OR ( TITLE-ABS-KEY ( mobile* W/2 ( based OR application* OR intervention* OR device* OR technolog* ) ) ) OR ( TITLE-ABS-KEY ( ( remote OR virtual OR digital ) W/2 ( evaluation* OR consult* OR management OR assessment OR clinic* OR intervention ) ) ) OR ( TITLE-ABS-KEY ( "tele referral" OR tele-referral OR telereferral ) ) OR ( TITLE-ABS-KEY ( telemedic* OR tele-medic* OR "tele medic*" ) ) OR ( TITLE-ABS-KEY ( tele-assistance OR "tele assistance" OR teleassistance ) ) OR ( TITLE-ABS-KEY ( "virtual medicine" ) ) OR ( TITLE-ABS-KEY ( telehealth OR tele-health OR "tele health" ) ) OR ( TITLE-ABS-KEY ( telecare OR tele-care OR "tele care" ) ) OR ( TITLE-ABS-KEY ( tele-rehab* OR "tele rehab*" OR telerehab* ) ) OR ( TITLE-ABS-KEY ( telediagnos* OR tele-diagnos* OR "tele diagnos*" ) ) OR ( TITLE-ABS-KEY ( telemonitor* OR tele-monitor* OR "tele monitor*" ) ) OR ( TITLE-ABS-KEY ( e-consultation* OR "e consultation*" OR econsultation* ) ) OR ( TITLE-ABS-KEY ( teleconsultation* OR "tele consultation*" OR tele-consultation* ) ) OR ( TITLE-ABS-KEY ( telecommunication* ) ) OR ( TITLE-ABS-KEY ( teleconferenc* ) ) OR ( TITLE-ABS-KEY ( videoconferenc* OR "video conferenc*" ) ) OR ( TITLE-ABS-KEY ( hotline* ) ) OR ( TITLE-ABS-KEY ( "remote consultation*" ) ) ) AND PUBYEAR > 2011 AND PUBYEAR < 2025 | 2,491 results |

# Summary of Clinical Trial Registries Searched and Results.

1. International Clinical Trials Registry Platform: (ICTRP) Searched condition, intervention and title 1 Jan 2012 – 12^th^ Jan 2025 start date trial. Results: 46
2. US Clinical Trials Register: (Clinicaltrials.gov). Searched condition, intervention and other terms 1 Jan 2012 – 12^th^ Jan 2025 start date trial. Results: 292
3. EU clinical trials register (EUCTR). Searched as one text block 1 Jan 2012 - 12 Jan 2025 start date trial. Results: 353
4. UK clinical study registry (ISTRCN) in Searched keyword/text search 1 Jan 2012 – 12 Jan 2025 start date trial. Results: 643

# Search terms for

## Each concept:

screen* OR evaluat* OR monitor* OR assess* OR identif* OR complication* OR apprais* OR detect* OR interpret* OR diagnos* OR recogni* OR attribut* OR intervention*

(encephalopath* OR concussion* OR concussive OR neurotrauma*) OR ((brain OR head OR cerebral) AND (injury OR injuries OR trauma OR anoxic OR anoxia OR hypoxia))

app OR apps OR online OR web OR internet OR digital* OR phone* OR telephone* OR smartphone* OR cellphone* OR smartwatch* OR "mobile health" OR mhealth OR m-health OR ehealth OR e-health OR emental OR e-mental OR remote OR virtual OR digital OR "Tele Referral" OR "Tele-Referral" OR telereferral OR telemedic* OR tele-medic* OR "tele medic*" OR tele-assistance OR "tele assistance" OR teleassistance OR "Virtual Medicine" OR telehealth OR tele-health OR "tele health" OR telecare OR tele-care OR "Tele Care" OR tele-rehab* OR "tele rehab*" OR telerehab* OR telediagnos* OR tele-diagnos* OR "tele diagnos*" OR telemonitor* OR tele-monitor* OR "tele monitor" OR e-consultation* OR "e consultation*" OR econsultation* OR teleconsultation* OR "tele consultation*" OR tele-consultation* OR telecommunication* OR teleconferenc* OR videoconferenc* OR "Video Conferenc*" OR hotline*

## ICTRP, Clinicaltrials.gov, EUCTR search:

((screen* OR evaluat* OR monitor* OR assess* OR identif* OR complication* OR apprais* OR detect* OR interpret* OR diagnos* OR recogni* OR attribut* OR intervention*) AND ((encephalopath* OR concussion* OR concussive OR neurotrauma*) OR ((brain OR head OR cerebral) AND (injury OR injuries OR trauma OR anoxic OR anoxia OR hypoxia)))) AND (app OR apps OR online OR web OR internet OR digital* OR phone* OR telephone* OR smartphone* OR cellphone* OR smartwatch* OR "mobile health" OR mhealth OR m-health OR ehealth OR e-health OR emental OR e-mental OR remote OR virtual OR digital OR "Tele Referral" OR "Tele-Referral" OR telereferral OR telemedic* OR tele-medic* OR "tele medic*" OR tele-assistance OR "tele assistance" OR teleassistance OR "Virtual Medicine" OR telehealth OR tele-health OR "tele health" OR telecare OR tele-care OR "Tele Care" OR tele-rehab* OR "tele rehab*" OR telerehab* OR telediagnos* OR tele-diagnos* OR "tele diagnos*" OR telemonitor* OR tele-monitor* OR "tele monitor" OR e-consultation* OR "e consultation*" OR econsultation* OR teleconsultation* OR "tele consultation*" OR tele-consultation* OR telecommunication* OR teleconferenc* OR videoconferenc* OR "Video Conferenc*" OR hotline*)

## ISTRCN search:

(screen* OR evaluat* OR monitor* OR assess* OR identif* OR complication* OR apprais* OR detect* OR interpret* OR diagnos* OR recogni* OR attribut* OR intervention*) AND (encephalopath* OR concussion* OR concussive OR neurotrauma*) OR ((brain OR head OR cerebral) AND (injury OR injuries OR trauma OR anoxic OR anoxia OR hypoxia)) AND (app OR apps OR online OR web OR internet OR digital* OR phone* OR telephone* OR smartphone* OR cellphone* OR smartwatch* OR "mobile health" OR mhealth OR m-health OR ehealth OR e-health OR emental OR e-mental OR remote OR virtual OR digital OR Tele OR e-consultation* OR "e consultation*" OR econsultation* OR videoconferenc* OR "Video Conferenc*" OR hotline*)

# Definitions

| Acquired Brain injury (ABI) | Damage to the brain that occurs after birth from a traumatic or non-traumatic event.  ABI is **not** related to a congenital disorder or degenerative disease, such as Alzheimer’s Disease, Multiple Sclerosis or Parkinson’s Disease. | Ontario brain injury association 2020 – referenced by the Ontario Neurotrauma foundation[4] |
| --- | --- | --- |
| Traumatic Brain Injury (TBI) | Damage to the brain caused by a traumatic event such as a blow to the head, a fall, a motor vehicle or sports related injury. | Ontario brain injury association 2020 – referenced by the Ontario Neurotrauma foundation[4] |
| Non-Traumatic Brain injury | **D**amage to the brain caused by illness such as meningitis or encephalitis, oxygen deprivation (anoxia) or stroke. (stroke will not be included in our study unless as part of a larger ABI cohort) | Ontario brain injury association – referenced by the Ontario Neurotrauma foundation[4] |
| Concussion | **The acute neurophysiological event related to blunt impact or other mechanical energy applied to the head, neck or body (with transmitting forces to the brain), such as from sudden acceleration, deceleration or rotational forces. Concussion can be sustained from a motor vehicle crash, sport or recreational injury, falls, workplace injury, assault or incident in the community.** | **Concussion Ontario: living concussion guidelines[5]** |
| Screening | Screening is the systematic application of a test or enquiry to  identify individuals at sufficient risk of a specific disorder to  warrant further investigation or direct preventive action,  amongst persons who have not sought medical attention on  account of symptoms of that disorder | Journal of Medical Screening : The Definition of Screening (2001) [6] |
| Assessment | Assessment refers to an evaluation of a condition based on the patient's subjective report of the symptoms and the examiner's objective findings, including data obtained through laboratory tests, physical examination, medical history, and information reported by family members and other health care team members. Assessment is a critical step in providing care to those who have a history of TBI by helping providers determine the extent of ones' TBI history, co-occurring mental health symptoms, and other sequelae. For information on interventions related to these screening and assessment areas please visit our Interventions page. | MIRECC TBI Toolkit – Screening and Assessment - [7] |
| Mild Traumatic Brain injury (mTBI) | Mild traumatic brain injury (TBI) is diagnosed when, following a biomechanically plausible mechanism of injury and one or more of the criteria (i-iii) listed below are met: i. One or more clinical attributable to brain injury. ii. At least 2 acute symptoms and at least one clinical or laboratory finding attributable to brain injury. iii. Neuroimaging evidence of TBI, such as unambiguous trauma-related intracranial abnormalities on computed tomography or structural magnetic resonance imaging (Criterion 5). | American Congress of rehabilitation medicine 2023. [8] |
| Moderate to Severe ABI | Patients are classified as having a mild, moderate or severe ABI according to their level of consciousness at the time of initial assessment. A moderate to severe ABI involves an alteration in consciousness. Common measures of severity include the GCS, the duration of loss of consciousness (LOC), and the duration of PTA. See Table below. | Ontario Neurotrauma Foundation referencing the ERABI tool. [4] |
| eHealth | “The use of information and communications technology in support of health and health-related fields” | WHO 2019 Guidelines on digital health [9] |
| mHealth | “The use of mobile wireless technologies for health” | WHO 2019 Guidelines on digital health [9] |
| Digital health | “The broad scope of digital health includes categories such as mobile health (mHealth), health information technology (IT), wearable devices, telehealth and telemedicine, and personalized medicine.” | FDA reports, what is digital health? (2020) [3] |
| Telemedicine | “The use of telecommunications and virtual technology to deliver health care outside of traditional healthcare facilities” | WHO 2019 Guidelines on digital health [9] |

Screening: Exclusion and inclusion criteria

**The inclusion criteria were all studies that:**

1. Were published in English (the language of the study authors)
2. Focused on digital health tools [9,10] for screening, evaluating, or monitoring ABI symptoms (either solely or as part of a larger patient cohort).
3. Reported primary research findings.
4. Were published between January 2013 and December 2024 inclusive. The decision to limit the search to this timeframe was informed by a preliminary search, which found that digital technologies predating 2013 were largely obsolete or incompatible with current digital platforms and clinical standards [9,10]; This approach has precedent in similar digital health scoping reviews [11–13]

**The exclusion criteria were studies that:**

1. Did not specifically address ABI or solely focus on participants with stroke.
2. Focused on non-digital methods of assessment without integrating digital tools, or when digital tools were not the focus of the evaluation.
3. Were narrative or systematic reviews, meta-analyses, editorials or opinion pieces lacking original research data.
4. Were descriptive, protocols only or still in development, i.e. they did not involve assessment of real human participants with ABI.
5. Did not have accessible full texts.
6. Involved bespoke clinical devices that were not off-the-shelf or readily available digital technologies as defined by the FDA and WHO guidelines on digital health and consumer grade devices [3,9]. Examples of technologies and tools that do not fit these definitions could include: digital intracranial pressure monitors, digital radiology devices such as MRI scanners, electroencephalograms, robotic gait aids, digital force plates etc.

**References:**

1. Rees L, Marshall S, Hartridge C, Mackie D, Weiser M. Cognitive interventions post acquired brain injury. Brain Inj 2007 Jan;21(2):161–200. doi: 10.1080/02699050701201813

2. Singh R-J, Chen S, Ganesh A, Hill MD. Long-term neurological, vascular, and mortality outcomes after stroke. Int J Stroke 2018 Oct;13(8):787–796. doi: 10.1177/1747493018798526

3. FDA. What is Digital Health? 2020.

4. Pavlina Faltynek MSc, Robert Teasell MD FRCPC. ERABI Clinical Guidebook - Introduction to Moderate to Severe Acquired Brain Injury. 2019. Available from: https://erabi.ca/wp-content/uploads/2019/09/Ch1_Introduction-to-ABI.pdf

5. Marshall S., Lithopoulos A., Curran D., Fischer L., Velikonja D., & Bayley, M. Living Concussion Guidelines: Guideline for Concussion & Prolonged Symptoms for Adults 18 years of Age or Older. concussionontario; 2023. Available from: https://concussionsontario.org

6. Wald NJ. The definition of screening. J Med Screen 2001 Mar 1;8(1):1–1. doi: 10.1136/jms.8.1.1

7. Rocky Mountain MIRECC. TBI Toolkit Screening and Assessment. Rocky Mountain MIRECC; 2024 Dec. Available from: https://www.mirecc.va.gov/visn19/tbi_toolkit/screening-and-assessment.asp#assessment

8. Silverberg ND, Iverson GL, Cogan A, Dams-O-Connor K, Delmonico R, Graf MJP, Iaccarino MA, Kajankova M, Kamins J, McCulloch KL, McKinney G, Nagele D, Panenka WJ, Rabinowitz AR, Reed N, Wethe JV, Whitehair V, Anderson V, Arciniegas DB, Bayley MT, Bazarian JJ, Bell KR, Broglio SP, Cifu D, Davis GA, Dvorak J, Echemendia RJ, Gioia GA, Giza CC, Hinds SR, Katz DI, Kurowski BG, Leddy JJ, Sage NL, Lumba-Brown A, Maas AIr, Manley GT, McCrea M, Menon DK, Ponsford J, Putukian M, Suskauer SJ, Van Der Naalt J, Walker WC, Yeates KO, Zafonte R, Zasler ND, Zemek R. The American Congress of Rehabilitation Medicine Diagnostic Criteria for Mild Traumatic Brain Injury. Arch Phys Med Rehabil 2023 Aug;104(8):1343–1355. doi: 10.1016/j.apmr.2023.03.036

9. Jandoo T. WHO guidance for digital health: What it means for researchers. Digit Health 2020 Jan;6:2055207619898984. doi: 10.1177/2055207619898984

10. Guardado S, Karampela M, Isomursu M, Grundstrom C. Use of Patient-Generated Health Data From Consumer-Grade Devices by Health Care Professionals in the Clinic: Systematic Review. J Med Internet Res 2024 May 31;26:e49320. doi: 10.2196/49320

11. Børtveit L, Dechsling A, Sütterlin S, Nordgreen T, Nordahl-Hansen A. Guided Internet-Delivered Treatment for Depression: Scoping Review. JMIR Ment Health 2022 Oct 4;9(10):e37342. doi: 10.2196/37342

12. Su Z, Zhang L, Lian X, Guan M. Virtual Reality–Based Exercise Rehabilitation in Cancer-Related Dysfunctions: Scoping Review. J Med Internet Res 2024 Feb 26;26:e49312. doi: 10.2196/49312

13. Bergschöld JM, Gunnes M, Eide AH, Lassemo E. Characteristics and Range of Reviews About Technologies for Aging in Place: Scoping Review of Reviews. JMIR Aging 2024 Jan 22;7:e50286. doi: 10.2196/50286
