# Supplementary material for: Digital Innovations for Clinical Assessment in Acquired Brain Injury: Scoping Review
Source: J Med Internet Res. 2025 Nov 5;27:e73331. doi: 10.2196/73331 (PMC12631094; doi:10.2196/73331)
Supplement: Multimedia Appendix 3 [file jmir_v27i1e73331_app3.docx]

| **Supplemental Table Outlining Data Charting:** | | | | | |
| --- | --- | --- | --- | --- | --- |
| **Theme & Assessment** | **Author (Year)** | **Product, Methodology & Included Population** | **Outcome Measures** | | **Main Findings** |
| **TBI Detection or Screening** | | | | | |
| **Smartphone or Tablet Based Tools** | | | | | |
| **Speech analysis** | Falcone M, (2013) (1) | Library for Support Vector Machines (LIBSVM), Cross Sectional Study, athletes mTBI (n=7) and Healthy Controls (n=98) | Sensitivity in mTBI screening vs expert assessment | | Platform was 98% accurate in detecting mTBI vs expert screening. Describes performance metrics, no statistical inference. |
|  | Yadav N, (2016) (2) | Sphinx Toolkit, Cross Sectional Study, youth athletes with mTBI (n = 95) and healthy controls speech recorded and analysed (n = 486) | Timing and frequency differences in speech parameters in mTBI vs controls | | SPHINX detected significant timing and frequency differences in TBI cohort vs controls, (P <0.5) ROC AUC: 0.73, No confidence indices or effect sizes. |
| **Passive sensor analysis** | Shelke S. (2021) (3) | TBI2Vec; Cohort Study – mTBI (n=19) and healthy controls (n = 179) | Sensitivity in mTBI screening over time vs Healthy controls | | The TBI2Vec model was sensitive at identifying mTBI, most accurate at 2d & 12 h post injury. F-beta (0.5) score of 83.0%, True Negative Rate of 96.0% and False Negative Rate of 33.0%. |
| **Screening exam** | Wilkerson GB. (2020) (4) | Flanker Test – (proprietary app adapted for tablet), Cross Sectional Study, athletes - mTBI (n=15) and healthy controls (n = 15) | Test-retest reliability (internal consistency coefficient and inverse efficiency index) for reaction times/response accuracy, correlation with symptoms on wellness survey in mTBI Cohort | | App-based Flanker Test reliable for reaction/ response times (ICC = 0.797); scores correlated with reported symptom burden (OR = 8.00, 90% CI [1.99,32.20]). |
| **Battery of tests with eye tracking** | Fischer TD, (2016)(5) | Unspecified program, iPad-based pro-point, anti-point testing and King-Devick (KD) ® testing, cross sectional study, Ed admitted mTBI cohort (n = 11), orthopaedic controls (n=7) and healthy controls (12) | Response time, sensitivity screening for mTBI vs standard assessment; differences in TBI-C, orthopaedic cohort and CONTROLS Performance measures of Pro-point tasks (initiation time [IT] and reaction time [RT]), Anti-point tasks (executive function testing), KD (oculomotor, vision and cognitive testing) tasks, as well as standardized assessment of concussion screen (SAC) and sensitivity in identifying mTBI cohort vs controls. | | mTBI group was significantly slower on Pro-point tasks, compared with the healthy group, as well as Orthopaedic group, for both IT (83 msec, t[27]= 5.96, p<.001 and 47 msec, t[27]= 2.95, p=.006, respectively) and RT (164 msec, t[27]= 9.50, p<.001 and 90.5 msec, t[27]= 4.55, p=.001; respectively), mTBI group was significantly slower on Anti-point compared with the healthy and orthopaedic group (124 msec, t[27]= 9.14, p<.001 and 91 msec, t[27]= 5.77, p<.001; respectively) and RT (190 msec, t[27]= 16.57, p<.001 and 124.57 msec, t[27]= 9.27, p<.001; respectively), There were significantly reduced SAC scores in mTBI group compared to healthy (t[16]= -2.61, p=.019) and the Orthopaedic group (t[16]=-2.21, p=.041).Performance on the KD task revealed no significant differences among the three groups (p=.72)Anti-point performance yielded the greatest AUROC (0.98; 95% CI= [0.96, 1.00]) suggesting it highly sensitive and specific for mTBI. |
| **Battery of screening tests** | Yang S, (2017) (6) | Brain Check™ (digital version of 6 neurological tests), cross sectional study, mTBI (n = 30), pain-matched controls (n = 30) and healthy controls (n = 538) | Validity of a composite of digital cognitive tests (Flanker test, digits symbol substitution test, Stroop task, trail making task and novel balance, coordination and recall tests) compared to standard ED assessments of TBI.  Sensitivity and specificity of Brain Check in differentiating TBI from controls | | The composite score distinguished TBI from non-TBI individuals with high sensitivity (83%) and specificity (87%) |
| **Computer or Web Based Tools** | | | | | |
| **Online survey** | Lequerica AH, (2018) (7) | OSU-BID - Cross Sectional Study - mTBI (n=95) and healthy controls (n = 170) | Completion rate; correlation of reported TBI parameters with RPQ, PRQS, CSS | | OSU-BID had an 89.4% completion rate; Worse TBI correlated with RPQ [P= .011] and TBI within previous 2 years correlated with RPQ cognitive subscales [P = .007]. |
|  | Gardner RC, (2020) (8) | OSU TBI-ID - Cross Sectional Study – TBI (n=49) and uninjured cohort (n= 118) of whom some had mild cognitive impairment | Reliability of online survey compared to in person interview; reliability compared to NACC-UDS screen | | OSU-TBI-ID was reliable compared to interview (κ 0.66–0.73; ICCs 0.68–0.81); NACC-UDS screen demonstrated fair reliability (κ 0.43) and 50% false negative rate vs interview. |
|  | Sullivan KA, (2024) (9) | Qualtrics™, OSUTBI-ID Cross Sectional Study – TBI (n=94) and uninjured cohort (n=62) | Completion rate, correlation of indices of TBI severity with symptom scales (inc. PCSS). | | High completion rate (94% of responses included), indices of severity correlated with PCSS and other symptom scores (P’s < 0.05, small–medium effect). |
| **Computer assisted telephone survey** | Cuthbert JP, (2016) (10) | OSU-BIIM CATI, Cohort Study TBI (n=94) and healthy controls (n = 100) | Retest reliability for cumulative, severity, and age-related indices of injury | | Reliable for indices of most severe TBI (OI= 0.62-0.78; k = 0.50-0.62), strong for number of TBIS with LOC > 30 mins (OI = 0.70 κ = 0.72) moderate to poor reliability for cumulative and age indices: (OI = 0.06 8 to 0.49; κ = 0.32 - 0.34) |
| **Screening test and survey** | Schatz P, (2013) (11) | ImPACT (online version), cross sectionally study, athletes, mTBI (n=118) and healthy controls (n=118) | Sensitivity and specificity in screening for mTBI in both symptomatic and asymptomatic athletes. | | In symptomatic concussed athletes, the impact subscales had 91.4% sensitivity and 69.1% specificity, the PLR was 2.95:1 and NLR 0.12.1 For asymptomatic athletes suspected of hiding their concussion, data from ImPACT yielded 94.6% sensitivity and 97.3% specificity, the PLR was 36:1 and NLR 0.03:1. |
| **Battery of assessments, questionnaire and voice analysis** | Rice V, (2019) (12) | Automated Neuropsychological Assessment Metric4 (ANAM4), Voice Analysis software (VA) (also included assessment of force plate and cerebral blood flow findings.), Cross sectional study, military, mild to moderate TBI (n=30) and healthy controls (n =53) | Comparison findings in TBI and control cohort of on neurocognition and mood testing through ANAM, cerebral blood flow using a Brain Acoustic Monitor (BAM), voice changes using Voice Analysis software (VA), and force plate balance measures. Analyse odds of predicting TBI with one or composite of parameters., | | Adjusted odds ratio for predicting TVI were 0.44 (95% CI.28 to.67) for *mean of ratings for the Vigor adjectives in the ANAM4 Mood Scale*, 2.38 (95% CI 1.16 to 4.91) as well as 2 force plate parameters. When authors assumed 15% pre-test probability, the 3 predictors yielded a predictive value of 75.7% with any two or more measures being positive, and a probability of 2.3% with zero measures being positive. (12) |
| **Speech & facial expression analysis** | Schultebrauck K, (2021) (13) | Parselmouth ™, Openface™, Deepspeech and machine learning (ML) models inc. XGBoost, Cross sectional study of Trauma Survivors with mTBI (n=55) and without (n=26) | Correlation of variety of ML based video and audio analysis of speech quality and content, facial features, and movement with WebNeuro ™ cognitive battery | | All analysis tools showed a strong correlation between abnormal speech/expression findings and multiple subdomains of cognition identified on WebNeuro testing including motor coordination (R2 = 0.52), processing speed (R2 = 0.42), emotional bias (R2 = 0.52),, sustained attention (R2 = 0.51), controlled attention (R2 = 0.44), cognitive flexibility (R2 = 0.43), cognitive inhibition (R2 = 0.64), and executive functioning (R2 = 0.63). XGBoost algorithm achieved high discriminatory accuracy for predicting the different cognitive domains using cross-validation (R2 ranged from 42 to 69%, MAPE ranged from 0.024 to 0.076). |
| **VR platform with eyewear** | | | | | |
| **Screening exam** | Sarker P (2022) (14) | HTC Vive Pro, Sranipal SDK, Cohort study, mTBI (n = 23) and healthy controls (n = 228) | Accuracy of different eye tracking models in detecting TBI Cohort vs Controls; sensitivity compared to vestibular oculomotor screen (VOMS) screen. | | Bidirectional LSTM model detected concussion with 92.3% accuracy, 73.91 % sensitivity and 94.29% sensitivity compared to VOMS., |
| **Telemedicine Based Tools** | | | | | |
| **Acute mTBI screening** | Vargas BB, (2017) (15) | VGo™ Telemedicine Robot; cohort Study, athletes, suspected sports related mTBI (n= 11) screened from 123 participants. | Agreement of telemedicine screen for sports related mTBI vs on-field screen (SAC, K-D and mBESS scores) and return to field of play (RFP) decisions. | | Agreement of face to face and tele-concussion SAC 100% of the time (6/6; 95% CI 54%–100%); Mean (SD) difference in K-D times was 0.7 (1.4) seconds. K-D times were within a 3-second difference 100% of the time (11/11; 95% CI 72%–100%). Remote and sideline mBESS scores were within 3 points 100% of the time (6/6; 95% CI 54%–100%). RFP decisions in, agreement 100% of the time (11/11; 95% CI 72%–100%). |
| **Symptom Assessment or Monitoring** | | | | | |
| **Smartphone or Tablet Based Tools** | | | | | |
| **EMA for symptom monitoring** | Sufrinko, A. M, (2019) (16) | Ilumivu ™ System -app, Cohort Study, athletes with mTBI (n=20), | Response rate, correlation with time of recording, PCSS, VOMS, cognitive screening, and recovery time. | | Mean EMA response to prompt rate of 52.4% (SD= 29.3), and 50.4% per participant no correlation between number of prompts and response rate (p= .77), symptoms were rated lower in the morning (P < .001), response rate reduced over time (OR= .91, 95% CI:0.87-0.94, P < .001), symptoms reported correlated with PCSS, VOMS and recovery time (p < .01). EMA responses more predictive of recovery than PCSS (B= 2.20, 95% CI: 0.38-4.03, P= .021). |
|  | Liu K, (2022) (17) | Healthstories Online™ -app, Cohort Study, veterans with mild to severe TBI (n = 49). | Completion rate, correlation of EMA symptom findings with HIT-6 scores | | EMA responses averaged of 2.5 entries per week, strong correlation with pain rating and average headache impact rating HIT-6 (r = 0.78 [P <.01] & r = 0.79 [P < .001]). |
|  | Ezekiel L, (2023) (18) | Proprietary Android App, Cohort Study; TBI (n=7), | Usability scores (SUS) and interview feedback, sensor reliability | | Participants reported good usability SUS 82.5, range 62.5–92.5; sensors found to be unreliable at capturing activity; 3 participant sensor results were suitable for assessment of Kappa statistics (K = 0.412, CI: 0.156–0.667; K = 0.315, CI = 0.128–0.503). |
|  | Ezekiel L, (2024 ) (19) | ThinkAloud™ with Fitbit actigraphy sensor; Qualitative study; TBI (n=9) | Themes from participant & therapist interviews, mean completion rate | | Themes from interviews included attending to experience, making sense of data, challenges in using data, relationship between fatigue and activity, implications for daily life, perceived benefits of self-monitoring, reviewing data in relation to understanding fatigue. Average of 78% (SD = 20) completion rate of EMA surveys. |
|  | Lazeron-Savu E, (2024) (20) | PsyMate™, Cohort Study – TBI (n = 10), | Compliance and completion rate, changes in fatigue (DMFS) score at week 6, qualitative feedback | | 70% of participants used app, of whom 71% gave improved DMFS scores at week 6; EMA completion rate averaged 56% with no significant reduction in responses (p = .99); in interviews it was reported to be useful for identifying triggers. |
| **EMA for psychological symptoms** | Juengst SB, (2015) (21) | iPerform ™, Cohort Study – mild to severe TBI (n=20) | Compliance with EMA prompts, satisfaction, and useability rating. Correlation between Symptom findings (e.g. GAD7 and PHQ 9) | | Mean EMA completion rate was 73.4%; Participants reported high satisfaction with smartphone applications (6.3 of 7) and ease of use easy to use (6.2 of 7). Comparison of assessments obtained via telephone-based interview and EMA demonstrated high correlations with PHQ-9 & GAD-7 (r = 0.81–0.97). |
|  | Juengst SB, (2019) (22) | iPerform ™ - cohort study, mild to severe TBI (n=18) | Variability in symptoms across time | | Reporting of all emotional, fatigue and associated symptoms fluctuated significantly across time (Z scores ranged 2.93 to 2.84; p = .002). |
|  | Juengst SB. (2023) (23) | BAST-mHealth- cohort study, mild to severe TBI (n = 52) and healthy controls (n = 12) | Compliance with EMA prompts, agreement with “full” BAST scores | | 79.7% of participants completed >/= 80% of EMA prompts; no significant difference in results vs standard BAST (p >0.05), significantly higher ratings of negative effects, executive dysfunction and fatigue vs controls (p <.05). |
|  | Little JR, (2017)(24) | "mCare" App – Randomized control trial, TBI (n = 183) | Wellbeing score (GWS) and case management quality scores (CMQQ) in treatment (mCare) vs control (SOC) arm | | Both groups had a statistically significant change in GWS (−2.2 (SE = 1.0; t = −2.1; p = .04) no significant difference identified between groups, No significant change in CMQQ in either control or treatment arm. |
|  | Forster SD, (2020)(25) | MovisensXS ™ - cohort study ABI (n=15) inc. TBI, stroke, encephalitis etc. | Compliance rate, response variation over time, correlation between compliance and demographics. | | Mean EMA completion rate was 71.4%; there was significant reduction in compliance associated with frequency of testing days (b= −2.2, SE= 1.16, t (88) = −1.88, p=.06); compliance not associated with age, depressive symptoms, or impairments (mean fluctuation measured as root mean square = -0.37 – 0.28). |
|  | Rabinowitz A, (2021)(26) | RealLife Exp.™ (LifeData Systems), Cohort study mild to severe TBI (n = 23), | Response rate, association of various activities with reported affect (e.g. on PANAS, etc.) | | 61% of participants responded at least 2/3rds of the time. Higher response rate associated with intact episodic memory (q = .57, p = .005) years of education (q = .35, p, .05), association of activities with affect varied by respondent, positive affect ratings were more likely while doing productive activity (p<0.05). |
|  | Rabinowitz A, (2024)(27) | RealLife Exp. ™ (LifeData Systems), cohort study severe TBI (n=39) | Response rate | | Mean EMA completion rate was 65%; response rate correlated negatively with the number of weeks enrolled (β=−0.0142, p=.0354), positively with education (β= 0.0549, p=.0108), with a marginally significant correlation to memory (β= 0.008, p=.0511). |
|  | Sherer M (2024)(28) | Mood Tracker app, (iLumivu™), Randomized Control Trial, mild to severe ABI (n = 127) | PHQ-9 and GAD-9 scores in treatment (receiving EMA) vs control (SOC) arm, compliance rate | | No significant difference in emotional distress scores between treatment and control groups (p = .39 %); mean EMA response rate was 80%. |
| **Function +/- quality of life survey** | Gvozdanovi AC, (2022)(29) | Vinehealth, Cohort Study, Brain Tumour (n=6), | Completion rate, participant feedback | | Completion rates (54% and 46%) were impacted by technical issues; 100% completion rates were seen when surveys were received correctly, participant feedback suggested they felt it improved their care. |
|  | Shukla D, (2023) (30) | GOSE (adapted), Cross Sectional Study – mild to severe TBI (n = 102), | Agreement with the interview-based GOSE scores | | Agreement of app-based questionnaire results compared with the interview results (k = 0.8, p<.01). |
|  | Lumetta K, (2023) (31) | FACT (novel survey), cross sectional study, paediatric, mTBI (n = 27) | Correlation of responses on FACT survey with SCAT and PCSS. | | The impact of symptoms on function recorded via FACT correlated strongly on linear regression with worse SCAT and PCSS scores (P <0.03). |
| **Survey on activity with actigraphy** | Huber DL, (2019 ) (32) | mHealth Survey (MS), Fitbit actigraphy, cohort study, mTBI (n = 25) and healthy controls (n=18) | Activity levels in TBI Cohort vs controls. Correlation between self-reported activity and actigraphy | | In the first 2 days post injury, there were fewer daily steps in mTBI vs controls, (P = .001), and actigraphy and self-reported measures indicated less moderate and vigorous physical activity in mTBI vs controls (P < .05). Moderate correlation between self-reported activity and actigraphy (r = 0.53, P=.006). |
|  | Wen PS. (2021) (33) | MOVES™ App, cohort study, military and civilian, mTBI (n = 7) and healthy controls (n = 5). | Study retention, satisfaction rate; correlation of activity with PART-O scores; activity rate in TBI cohort vs controls. | | Study had 75% retention rate, Perceived accuracy of the MOVES app was 90%, while the both TBI and healthy controls showed similar discrepancies between the PART-O and the MOVES (52% vs. 53%). |
| **Survey on sleep (+/- actigraphy)** | Lee MJ, (2021) (34) | MySleepScript™ App, Cohort study ABI (n=65) inc. TBI, stroke, structural brain pathology etc. | Time to completion,  Sleep quality scores (inc. PSQI) in TBI cohort vs other etiologies of ABI | | Mean completion time was 16 min (SD=5.4),  TBI cohort reported worse PSQI scores vs other ABI (mean 10.1 [SD=4.5] versus 7.4 [SD=4.7]; t=2.08, df=57, p=.04, respectively). |
|  | Morrow EL (2024) (35) | Actigraph GT9X™ activity monitor, cohort study, moderate to severe TBI (n = 45) and healthy controls (n = 47) | Accuracy in self-reporting in TBI cohort vs Healthy controls. Sleep duration, rate of "wakeups" vs sleep quality reports. | | Strong correlation between reported sleep duration and actigraphy in both groups, (NC: r = 0.51, p < .001, TBI: r = 0.59, p < .001, see Figure 1). No statistically significantly difference between reported sleep quality and actigraphy measurements in either group on MLR model (estimate = .162, t = 1.098, p =.272). Poor correlation in both groups between self-reported wakeups and actigraphy findings. (Controls: r = 0.11, p=.62, TBI: r= 0.044, p =.83). |
| **EMA and heart rate variability (HRV) monitoring** | Nabasny A, (2022) (36) | BAST mHealth™ and Elite HRV™ apps; Polar 10 HR sensor, Cohort study, Mild to severe TBI (n = 52) and healthy controls (n = 12) | Covariance between HRV and EMA neuro-behavioural (NB) symptom responses to bast mHealth survey (i.e. Negative Affect, Fatigue, Executive Dysfunction, Substance Abuse and Impulsivity Subscales). | | Cross-correlation coefficients (r ≤-. 30) were seen in 27.1-29.2% of participants for Negative Affect, Executive Dysfunction, and Fatigue; 20.8% of participants for Impulsivity, and 10.4% of participants for Substance Abuse.  In 2.0%−20.8% of participants there was positive cross-correlations (r ≥.30) across all subscales, 54.2%-87.5% of participants had no significant cross-correlations (−.30<r<.30). |
| **Predicting symptoms using sensor data** | Schultz LS (2024) (37) | Mindstrong Discovery™, cohort study, mild to severe TBI (n = 15) | Rate of uptake (consent to install app) and transfer of data. Exploratory outcome: predicting depression (PHQ9>10) compared to chance (shuffled dataset). | | 93.8% of subjects consented and installed app (90% CI = 0.698-0.998), of whom 93.3% had at least 1 data transfer; sensor analysis predicted depression greater than chance (P= .007, Mann–Whitney U). |
| **Computer or Web Based Tools** | | | |  | |
| **Computer assisted text message survey** | Suffoletto B, (2013) (38) | TIPS – software not named, Randomized control trial, mTBI (n = 43) | Changes in reported symptoms and findings on symptom burden scales (RPQ, and PHQ-9) in Intervention arm (Regular assessment and advice) VS control arm (Standard of care).  Response rate to text message survey and reported usefulness. | | Compared with the controls, intervention participants trended to lower odds of reporting headaches (OR = 0.38; 95% CI 0.07-1.99), concentration difficulty (OR = 0.32; 95% CI: 0.04-2.24), and irritability or anxiety (OR = 0.33; 95% CI: 0.05-2.35). There were trends of lower mean scores for headache (0.99 vs 1.19; P = .5), difficulty concentrating (0.88 vs 1.23; P = .2), and irritability/anxiety (1.00 vs 1.62; P = .06). There was no statistically significant difference seen. 78 % of the treatment group were followed up at 14 days, of whom here were high response rate to SMS symptom assessments (74-96%) and high satisfaction with the intervention (93% reporting it to be “at least somewhat useful”). |
|  | Anthony CA, (2015) (39) | Concussion Symptom Severity Score (CSSS), adapted for computer assisted text survey. Cohort study, mTBI (n = 14) | Repeatability coefficient of CSSS across time. | | CSSS were poorly repeatable across time (mean 23.6 day follow up, Repeatability coefficient was +/- 23.1). |
|  | Schoenfeld R, (2022) (40) | ConText study, mHealth adapted Post-Concussion Symptoms Inventory (mHealth-PCSI)  Cohort study, mTBI (n=31) | Correlation of mHealth PCSI with standard PCSI and standard  Retention rate and useability rating. | | There was a strong and positive correlation between PCSI and the mHealth tool (rs = 0.875, P < .000, n = 22).  Retention rate was 74% until symptom resolution, 42% to study completion. |
| **Online survey** | Karvandi E, (2024) (41) | Onlinesurveys.ac.uk, Cohort study, mTBI (n = 200) | Number of eligible patients, follow-up rates, survey response rates | | The 1^st^ survey had a 67% completion rate, 47.5% of participants completed all three; 99% responded that the survey was useful and acceptable, 98% responded they were happy with the format. |
|  | Shaikh N, (2021) (42) | BIST, Cross sectional study, mTBI (n = 114) | Internal construct validity, dimensionality, Rasch analysis findings | | BIST demonstrated acceptable model fit χ2(6) =3.8, p >.05), with good reliability (Person Separation Index = 0.84), and uni-dimensionality for screening mTBI symptoms and quantifying severity. No significant effects for sex or age were identified |
| **Computer-assisted telephone and online survey** | Wong AK, (2014) (43) | Computer adapted testing of community participation indicators (CAT CPT), cross sectional study, Participants with a variety of conditions (n=674) inc. TBI (n=40), stroke (n = 73), other neurologic disease (n = 47), spinal cord injury (n =73), non-neurologic condition (n = 442) | Completion rate of online vs telephone-based CAT CPI completion, factors associated with choice off online VS CATI | | CAT-CPI had a 34% completion rate, 61% of respondents chose telephone vs 39% choosing online, no significant difference in completion rate between online or telephone-based respondents, choice of modality predicted by demographic factors inc. age, gender, retirement status, length of hospital stays (x2=51.73, P=.001). |
| **Physical Examination** | | | |  | |
| **Smartphone or Tablet-Based Tools** | | | |  | |
| **Gait, balance or posture examination** | Rhea CK, (2017) (44) | AccWalker ™ App, Cohort study, military participants before and after blast exposure, mTBI (n=59) | Sensitivity to neuromotor changes after blast exposure | | Mean stride time was significantly longer post immediate exposure (6.35 ms longer) and at 72-96h post injury (5.41ms longer) when compared to pre-exposure testing (p<.001). |
|  | Rhea CK, (2022) (45) | AccWalker ™, Cross sectional study, military and civilian mTBI (n=62) and controls (n=154) | Movement variability (MV) on balance assessment, measured by a variety of parameters (such as variability of Max velocity [SD_MaxVel]) conditions (such as eyes closed or head shake) in mTBI cohort vs controls | | The app demonstrated a significant movement variability in on measures of SD_MaxVel in TBI cohort compared to controls on Linear mixed-effects modeling (p<.001), this effect was more marked on head shake compared to eyes open condition (p=.031). |
|  | Feigenbaum LA, (2019) (46) | CaneSense™ and mBESS, Cohort Study, athletes, mTBI (n =8) compared to pre-injury baseline assessments | Changes in post excursion concussion index (PECI - composite score based on Canesense SLS) and mBESS composite score, from pre and post injury assessments | | Significant change in the PCEI score (41.43 ± 15.53% vs. 87.41 ± 6.05%, p<.001); unanticipated improvement in mBESS (10.5 ± 4.87 errors vs. 16.5 ± 8.49 errors, p=.10). suggesting Canesense PCEI may be more sensitive. |
|  | Manor B, (2019) (47) | TeamStudy ™, cross sectional study, athletes, mTBI (n=66) | Correlation of high head trauma burden history and loss of conscious history (LOC) with findings of impaired balance | | Both a high TBI burden and LOC history correlated with increased stride time variability (F = 6.9, p=.005 and F = 4.5, p=.001; respectively) |
|  | Kis M, (2020) (48) | EQ balance™, & Sway Balance™, cross sectional study mTBI (n = 39) and healthy controls (n = 31) – split into 2 groups healthy balance (n = 44) and balance impaired (n =26) | Safety; agreement in balance findings between apps and difference in findings between sub-groups | | Both apps were safe, Average balance scores in statistical agreement between both apps across entire group, and sub-groups , on Deming and Spearman testing (p<.01); with strong consistency between results (ICC = 0.87 , p<.001) on post hoc analysis, balance scores in balance impaired cohort (EQ73.5, Sway 67.7) were worse than healthy balance group (EQ 87.0, Sway 90) on Mann Whitney U Testing (P<0.001). |
|  | Dummar MK, (2024) (49) | Sway Balance™, Cohort Study, mTBI (n = 20) and healthy controls (n = 20) | Difference in mBESS scores in TBI Cohort vs Healthy Controls, changes over time, correlation with sensory organization test (SOT) | | Sway™ did not detect significant difference in mBESS scores in TBI cohort vs controls, nor did findings change over time, [F (2,40) = .114, p=.89; F (2,40) = .276, p =0.60]. No correlation between mBESS scores & SOT scores (r = -0.317 to -0.062, p >.05). |
|  | Tirosh O, (2024) (50) | TelePhysio™, cross sectional study, mTBI (n= 11) and healthy controls (n=11) | Difference in postural sway metrics in TBI Cohort vs Healthy controls | | App identified significant differences sway metrics in TBI cohort vs controls on Anova testing (Group F = 5.615 to 7.058; all P values <.001). |
| **Pupillary light response (PLR) exam** | Carrick FR, (2021) (51) | BrightLamp Reflex ™, Retrospective review of clinic attendees with mTBI (n = 4999) and without mTBI (n = 22847) | Difference in PLR parameters in TBI Cohort vs non-TBI cohort | | Significant differences in multiple PLR parameters in TBI-Cohort vs non-TBI, maximal pupillary diameter was predicted by age, gender and mTBI status on multiple linear regression model (p=.000, R2 = 0.0834). |
|  | McGrath LB, (2022) (52) | PupilScreen™, Cross sectional study, severe TBI (n = 6) and healthy controls (n = 42) | Predictive value of abnormal PLR findings to identify TBI vs controls; and reliability within and between groups of assessors (doctors and nurses) | | App had a 93% accuracy, 94% sensitivity, 92% specificity, 92% positive predictive value, and 93% negative predictive value in identifying normal vs abnormal PLR curves. High within-group reliability (k = 0.85, CI not given), high interrater reliability (K = 0.75, CI 0.71, 0.79). |
|  | Maxin AJ, (2023) (53) | PupilScreen™, Cross sectional study, severe TBI (n=33) and healthy controls (n=132) | Differences in PLR findings on PupilScreen exam in TBI Cohort vs controls, the best performing combination of PLR findings, sensitivity compared to instrumented pupillometry measuring the proprietary neurological pupillary index (NPi) severity score | | PupilScreen™ app identified significant differences in PLR findings in TBI cohort vs controls in all PLR parameters. The top performing combination was maximum pupillary diameter, mean constriction velocity, maximum constriction velocity, and mean, dilation velocity (accuracy = 87%, sensitivity = 85.9%, specificity = 88%, AUC = 0.869 and F1 score = 0.85, on random forest model). This outperformed the NPi score (accuracy = 67.4%, sensitivity = 50.9%, AUC = 0.648, and F1 score = 0.567, respectively). |
|  | Dutta P, (2024) (54) | Reflex Pro™, Cross sectional Study, athletes, mTBI (n = 50) and healthy controls (n = 50), | Difference PLR findings in TBI cohort vs controls | | Reflex Pro™ identified significant differences in PLR parameters in TBI cohort vs Healthy Controls: average constriction speed (1.10 ± 0.15 vs 1.78 ± 0.12 mm/s; P=.001), maximum constriction speed (2.05 ± 0.26 vs 3.84 ± 0.28 mm/s; P=.001), average diameter (3.64 ± 0.12 vs 0.36 ± 0.05 mm; P=.001), maximum diameter (4.75 ± 0.17 vs 5.23 ± 0.16 mm; P=.001), and minimum diameter (2.75 ± 0.17 vs 3.64 ± 0.11 mm; P=.001), dilation release amplitude (0.54 ± 0.96 vs 0.36 ± 0.05 mm; P=.001) and latency (0.25 ± 0.05 vs 0.21 ± 0.02 s; P=.001). |
| **Computer or Web-Based Tools** | | | |  | |
| **Analysis of limb movement** | Mobbs A (2024) (55) | DeepLabCut ™, cross sectional study, moderate to severe TBI (n = 42) and healthy controls (n=5) | Agreement of Machine learning (ML) based analysis, and clinician rated limb movement abnormality during gait | | ML rating of abnormal gait strongly agreed with clinician scores on Anova testing (p<0.05). |
| **Telemedicine Platform** | | | |  | |
| **Adapted clinical exam** | Llamas-Rojas R (2023) (56) | Fugyl Meyer adapted for Telemedicine (FMA-TV), cross sectional study, ABI (n = 30) etiology not-specified) | Agreement between FMA-TV and in person FMA carried out at the same time, analysed through Cronbach’s Internal Consistency (CIC) and Weight Kappa Index (WKI) | | Substantial agreement between FMA-TV and in-person FMA - Exploratory and confirmatory factor analysis = 76.8% variance, CIC = 0.98, and WKI = 0.78 |
|  | Soria MZ, (2023) (57) | Orofacial Myofunctional Evaluation with Scores (OMES), Google Meet™, cross sectional study, moderate to severe TBI (n=23) | Reliability of telemedicine OMES with in-person OMES measured on interclass correlation coefficient (ICC) | | Excellent reliability between OMES scores from TMP and in person assessments (Mean difference was −0.04 CI: −1.12, 1.04; ICC ≥ 0.85). |
| **VR platform (with eyewear)** | | | |  | |
| **Balance or gait assessment** | Teel EF, (2015) (58) | VR Balance Module, Cross sectional study, mTBI (n = 21) and healthy controls (n = 60) | Correlation of VR balance scores and pressure plate findings with eyes open and closed conditions; comparison of VR balance scores in mTBI cohort vs controls | | VR based balance scores correlated strongly with pressure plate findings (correlations ranged from r= −.273 to −.704 for eyes open and from r= −.353 to −.876 for eyes closed conditions), mTBI cohort did worse on all VR scores on Anova testing (p<.01). |
|  | Robitaillea N, (2016) (59) | VrAI, Cross sectional study, soldiers, mTBI (n=6) vs healthy controls (n=6) | Difference in walking fluidity in TBI cohort vs health controls vs CONTROLS; subject feedback of useability and adverse outcomes | | Walking fluidity less dynamic in TBI cohort vs Healthy Cohorts, on Anova testing (p=.046). Test was mostly well tolerated, one subject suffered nausea. |
| **Visio-spatial assessment** | Painter DR, (2023) (60) | The Attention Atlas, Cohort study, ABI cohort (n= 12) inc. TBI, stroke, other and non-brain injured controls (n = 9) | Feasibility: subjective report of experience using Likert scale, usability ratings, comparison of visuospatial atypicality findings in ABI vs control cohort, Accuracy at identifying visuospatial neglect compared to pen-paper assessment. | | Median responses on experience reporting included a: “moderate” when asked about: the positive affect (median, 3.6; IQR, 0.9), competence (median, 3.5; IQR, 0.8), and flow (median, 3.1; IQR, 0.8), and “not at all” when asked about negative affect (median, 1.4; IQR, 0.4), median usability ratings were acceptable (median, 80; IQR, 13.1), Visuospatial atypicality was more prevalent in ABI cohort than in controls (χ2=19.46, P<0.001) Atypicality was greater among ABI cohort (Levene’s test L = 10.5, p=.004), VR testing identified all 3 cases of neglect identified on pen-and-paper testing & additional 3 patients with atypical visuospatial patterns that were not identified via traditional assessments |
| **Cognition or Memory** | | | | | |
| **Smartphone or Tablet-Based Tools** | | | | | |
| **Battery of assessments** | Rebchuk A, (2020) (61) | National institute of health TBI cognitive toolbox (NIHTB-CB), Retrospective review, mild TBI (n = 66), stroke (n = 63); patients with psychosis (n = 39) and healthy controls (n = 77) with cognitive assessment findings from previous studies | Estimated prorated scores of fluid and total cognition from standard NIHTB-CB carried out previously on study participants, Comparison of prorated and standard NIHTB-CB scores | | For fluid cognition, overall prorated scores were higher than standard scores (mean difference= þ4.5, SD= 14.3; p<.001; ICC= 0.86). For total cognition, overall prorated scores were higher than standard scores (mean difference= þ2.7, SD= 8.3; p<.001; ICC= 0.88). Significant in TBI group. (Difference 5.8, P <0.001, ICC 0.89). |
|  | Spreij LA, (2020) (62) | Digital Neuropsychologic assessment (d-NPA ™), cross sectional stud, ABI (n=120) inc. stroke and TBI, and healthy controls (n=159) | Completion rate of d-NPA i.e. 12 neurological assessments previously validated for paper-based testing: Rey Auditory Verbal Learning Test (RAVLT) immediate recall, Trail Making Test (TMT)part A and B, Cube Drawing, O-Cancellation, Clock Drawing, Star Cancellation, RAVLT delayed recall and recognition, Rey-Osterrieth Complex Figure (ROCF) copy, Verbal Fluency Letter, ROCF immediate recall, Digit Span forwards and backwards, Verbal Fluency Category, Stroop Color and Word Test Stroop), ROCF delayed recall, and the Wisconsin Card Sorting Test (WCST), Validation through comparing healthy cohort results to known paper-based test percentiles User ratings via interview Effect of tablet familiarity with scores | | d-NPA had a 94% completion rate among ABI and 100% completion among healthy controls, 34% of Healthy Controls scored in 10th percentile paper-based norms of half the tests, RAVLT (immediate recall, delayed recall, recognition), TMT A, Clock Drawing, Cube Drawing, ROCF copy, Verbal Fluency Letter, Verbal Fluency Professions, WCST number of completed categories, and the WCST failure to maintain set) Tablet use (hours/week) had no significant effect on test performance (e.g., RAVLT Immediate: F-change = 1.20, p = .276) 91% of participants found the d-NPA pleasant; 6% required adjustments (brightness/volume) . |
|  | Spreij LA, (2021) (63) | Digital Neuropsychologic assessment (d-NPA ™), Cross sectional study, ABI (n = 161) inc. stroke, TBI and tumour, and healthy controls (n= 91) | Differences in performance stability (i.e., moment-to-moment fluctuations) between ABI and healthy cohort in adapted cognitive battery (RVLT, TMT A+B and Stroop)  Comparison of performance stability measures vs conventional cognitive testing in differentiating ABI vs controls  Relation between performance stability and cognitive complaints in daily life in ABI cohort. | | Participants with ABI fluctuated more in all tests vs controls, e.g. for RAVLT immediate recall naming speed (F (1, 186) = 5.00, p = .027, η2 = .026).  4–15% of ABI cohort who initially scored within normal range on conventional scores demonstrated abnormal performance stability findings.  Neither performance stability or conventional cognitive score correlated with cognitive complaints. |
|  | Wallace SE, (2019) (64) | Standardized Touchscreen Assessment of Cognition (STAC), cross sectional study, moderate to severe TBI (n = 45) | Correlation of STAC with Montreal cognitive assessment (MoCA) and Cognitive Linguistic Quick Test CLQT; participant feedback on Post Study System Usability Questionnaire | | Moderate correlation with MOCA & CLQT; mixed comfort levels reported The MoCA and STAC had two areas with correlations greater than 0.5 (orientation, generative naming first letter), while the CLQT and MoCA had three areas greater than 0.5 (orientation, generative naming first letter and category). 60% of participants strongly agreed tablet-based testing was comfortable to use, and that they would use a tablet in future |
| **Novel or adapted assessment** | Chen PM, (2024) (65) | ImPACT-QT (abbreviated form of previously validated tool: ImPACT), cross sectional study, mTBI (n = 179) and comorbid “trauma” controls (n = 54) | Differences in Completion time between mTBI and healthy cohort, useability survey responses | | Mean test-time was 9.3 ± 2 minutes with 93% mTBI cohort had lower memory scores compared to controls (25 [IQR 7-100] vs 43 [26-100], P = .001) while attention (5 [1-23] vs 11 [1-32]) and motor scores (14 [3-28] vs 13 [4-32]) showed no Significant differences (p=81 and .755 respectively). 90% of survey respondents reported test easy to understand (no difference between groups, p = .203) |
|  | Mendez-Lopes M, (2024) (66) | SLAM™-based proprietary app, Cross Sectional Study, ABI (n = 10) including stroke, cerebral abscess, meningitis, tumour etc.) and healthy controls, (n = 10) | Time needed for ABI and control cohorts to complete AR-based tasks in vs healthy controls Usability and Acceptance Survey (UAS) responses and interview feedback. | | Compared to controls, the ABI cohort was slower at completing AR-based tasks (U= 23.00, Z= 2.04, p= .041, r = 0.45), showed a worse recall of the location of the objects (% Success: U= 90.00, Z= 3.42, p= .001, r = 0.76), and more attempts to complete tasks (% Attempts: U= 5.00, Z= 3.74, p < .001, r = 0.84). ABI cohort rated usability and acceptance positively, but significantly lower than healthy controls. (Usability: U= 94.00, Z= 3.38, p= .001, r = 0.75; enjoyment: U= 82.50, Z= 2.80, p= .005, r = 0.63) |
| **Memory training & testing** | Morrow EL (2024) (67) | Memi™, Memi, Twilio, Cohort Study, moderate to severe TBI (n =14) | Rate of session completion, System Usability Scale (SUS) scores, interview feedback | | Memi app had 98% completion rate i.e. 11.8 of 12 available sessions completed on average (SD = 0.4); the mean SUS score was 91.4 (SD 8.6) described as an A+ result on standardized scale; in interviews participants described the app as simple and easy to use, and helpful for memory. |
| **Computer or Web Based Tools:** | | | | | |
| **Task-based screening** | Canty, AL, (2014) (68) | Virtual Reality Shopping task (VRST) cross sectional study, severe TBI (n = 30) and healthy controls (n = 20) | Difference in prospective memory (PM) performance in VRST on event and time-based tasks Performance on VRST vs a standard prospective memory (PM) task (lexical decision PM task [LDPMT]) Sensitivity and specificity of identifying TBI cohort vs controls | | TBI cohort performed significantly poorer than controls on the ongoing component of VRST, t (49.44) = 3.54, p = .001, d = .92) Strong correlation between event based and total VRST tasks and LDPMT in TBI cohort (r = 0.657, 0.662 respectively, p<.001), VRST Total PM scores demonstrated (90%) and moderate sensitivity (76%) for identifying TBI cohort vs controls |
|  | Goverover Y, (2015) (69) | Actual Reality (AR), cross sectional study, TBI (n = 10) and healthy controls (n = 10) | Difference in cognitive scores (0-128) based on performance on task (a cumulative index based on time to competition, errors, need for cueing etc.), rate of step errors, total errors, and time to completion in TBI vs control cohort. | | Cognitive score for the TBI cohort (Median = 11, SD= 5.3) was significantly worse than that of healthy participants (M= 5.8, SD = 41) [F (1, 19) 5.9, p=.02, ε^2^ = 0.25], had more step errors (M= 25.5, SD = 19.8) vs Controls (M=11.3, SD = 8.9) (F(1, 19) = 6.2, p=.02, ε^2^ = 0.25); the control group made less total errors (M=6, SD¼ 4.1) than the TBI group (M = 13, SD = 7.6) [F(1, 19) = 6.5, p =0.02, ε^2^= 0.26] and had shorter completion time (M =678, SD = 285.7) the TBI group (M = 1789.6, SD = 759.4) (F(1, 19) 18.4, p=.001, ε^2^= 0.53). |
|  | Martínez-Pernía D, (2017) (70) | eAdventure, Serious Game authoring platform, Cross Sectional Study – TBI (n = 8) as well as their therapists. | Completion and error rates of a computer based naturalistic functional task, quantitative feedback through Technology Acceptance Model (TAM), and qualitative user feedback | | Participants had no commission, omission or preservation errors, therapists reported test was useful and easy to use (mean = 2.28, sd = 0.86 and (mean = 2.63, sd = 0.44) no significant difference in sub-scores measuring perceived usefulness and ease of use (p<.05). Major themes on interview included ease of use, ecological validity, script complexity (some suggested it is too simple), interface, and overlearning (participants suggested familiarity with task can affect outcomes. |
|  | Van Nostrand (2019) (71) | Proprietary programme testing single and divided attention task performance, Cohort study, mTBI (n = 33) and healthy controls (n = 159) | Consistency of performance (computational math score [CMS]) in single and divided attention conditions at 1, 6 and 12 weeks, effect of daily practice among a sub- group of 33 healthy controls, and sensitivity in identifying mTBI | | Individuals with mTBI demonstrated worse results, correlation consistent across all timepoints (ICC > 0.5 throughout), Linear improvement with daily practice among health control subgroup (single task: R2 = 0.89, divided-attention: R2 = 0.85), CMS’s were significantly lower in mTBI cohort vs controls under both single (456.0 ± 75.0; 532.9 ± 58.1; p < .001) and divided attention (417.7 ± 80.6; 480.2 ± 46.1; p < .001) conditions. |
|  | Nadler T, (2022) (72) | Internet bill paying task (IBPT), cross sectional study, inpatients with TBI (n = 42) and healthy controls (n=47) | Time and cues needed to complete IBPT, correlation with WebNeuro neurocognitive computerized battery, Semantic Verbal Fluency test (SVFT), Behavioural Assessment of the Dysexecutive Syndrome (BADS), Dysexecutive Questionnaire (DEX), and cognitive items of the Functional Independence Measure and Functional Assessment Measure (cognitive FIM/FAM). | | On IBPT the TBI cohort needed a median of 7.0 cues (IQR5 3.7–10), control needed a median of 1.0 cue (IQR5 0–2). Effect size was large for total score and executive subscale: (R = 0.63 & 0.59 respectively). IBPT strongly correlated with DEX (r = 0 .65, p < .01) and FIM/FAM scores (r = 0 .66, p < .01) and moderate to strongly correlated with the SVFT, BADS total score and execution score, and several sub tests of Web-Neuro battery (p<0.05). |
|  | Lencsés A (2024) (73) | Virtual Week, cross sectional study, mild to severe TBI (n = 18) and healthy controls (n = 18) | Prospective memory (PM)and retrospective memory (RM) task performance (proportion of correct responses and recognition of tasks respectively) in TBI and control group | | TBI cohort had significantly worse PM results (M = 0.43, SD = .30) vs controls (M = 0.86, SD = .11) (p <0.05) and worse RM performance (M = .72, SD = .25; & M = .95, SD = .06 respectively) (p<0.05). |
| **Battery of assessments** | Del Giovane M, (2023)(74) | Cognitron ™, Cohort study, Stage 1 (task selection): moderate to severe TBI (n=126), and healthy controls (n=84), Stage 2 (validation vs standardized testing) TBI (n=48), Stage 3 (clinical pilot) new TBI cohort (n=50) compared to normative dataset (n>130,000) | Stage 1: Sensitivity of cognitive tasks to discriminate TBI from control, Stage 2: Shared variance (determined using Canonical Correlation Analysis [CCA]) of battery of 8 online tasks with standardized cognitive tests: Montreal Cognitive Assessment (MOCA) and repeatable battery for the assessment of neuropsychological status (RBANS), Stage 3: reliability of detecting cognitive deficits in TBI cohort vs normative dataset | | Stage 1: 15 of 23 tasks showed significant discrimination (F > 4.0, all p values < 0.05), 8 optimal tasks selected, Stage 2: CCA identified two significant modes indicating shared variance of 8-task battery with MOCA and RBANS (mode 1 [predominantly memory based]: r = 0.86, p<.001; mode 2 [predominantly executive visuospatial skills]: r = 0.81, p=.02), Stage 3 Online tasks reliably detected deficits (17/17 tasks had significant t-values; e.g., Card Pair (summary): t = -3.88, p<.001 SRT (summary): t = -6.96, p<.001 Trail Making B (reaction time): t = -7.32, p<.001. |
|  | Pellinen (2024) (75) | Cogstate™ Brief Battery (CBB), multinational cohort study, focal epilepsy (n = 408) | Completion rate of cognitive battery (including simple reaction time, choice reaction time, 2D manipulations, picture completion, card pairs, trail making, Tower of London, paired associates learning), characteristics associated with non-completion | | CBB had a 61% completion rate; on multiple logistic regression: male participants were more likely to engage in testing (OR 2.14, 95 % CI 1.29 to 3.5, p <0.01), Black subjects were less likely (OR 0.45, 95 % CI 0.22 to 0.9, p=.02), primary. English speakers were more likely (OR 3.1, 95 % CI 1.21 to 7.96, p=.02), and those with a history of learning challenges were less likely (OR 0.69, 95% CI 0.49 to 0.97, p=.03). |
| **VR platform with eyewear** | | | | | |
| **Task based assessment** | Seton C, (2023)(76) | SeaHero Quest (SHQ), Cross sectional study, athletes, mTBI (n = 15) and healthy controls (n= 23) | Wayfinding performance (Distance to navigate to virtual end point on SHQ) in TBI and healthy cohorts. Correlation with Santa Barbara Sense of Direction Scale. The Santa Barbara Sense of Direction Scale (SBSOD). | | mTBI and control group self-reported wayfinding on the SBSOD was not significantly different (U = 140.50, p= .34) On SHQ wayfinding assessment, the control group traveled significantly shorter distances : (-274.28 [pixels], 95% CI:-412.39to -136.16) t [21]= -4.13, p<.001, 2-tailed, Cohen’s d= -1.74), mean duration was significantly shorter for the control vs mTBI cohort (-24.69 [seconds], 95% CI: -42.62to -6.75, t[21]= -2.86, p= .005, 2-tailed, Cohen’s d= -1.20). |
|  | Okahashi, S (2014)(77) | Virtual Shopping Task Revisited (VSTR) (inc. both cognitive and visual neglect assessment), Cross sectional study, ABI (n=7) inc. TBI, stroke, encephalitis), and healthy controls (n=6) | Completion rate of VRST cognitive and visual neglect assessment in ABI and control cohorts Correlation of VRST performance metrics (task completion time, hints needed, accuracy) with battery of validated tests on attention, memory and executive function, inc. Mini Mental State Exam (MMSE), Symbol Digit Modalities Test (SDMT), Simple Reaction Time Task (SRTT), Star and Letter Cancellation Tasks (SLCT), Rivermead Behavioral Memory Test (RBMT), Everyday Memory Checklist, Zoo Map Test and Dysexecutive Questionnaire (DEX) | | VSTR task completion rate was similar in TBI and control cohorts. TBI cohort took longer to complete tasks and required more navigational aids (No summary statistics given). Performance metrics reported to correlate with some attention, memory and cognitive tasks (summary statistics not given) |
|  | Teel EF, (2016)(78) | HeadRehab (Battery of VR modules testing navigation, attention, body reaction time (RT) and balance) – Cross Sectional Study, mTBI cohort (n=24) and healthy controls (n= 128). | Sensitivity and specificity in identifying cognitive & sensorimotor deficits TBI cohort compared to healthy controls | | Several VR modules were both sensitive and specific in identifying cognitive & sensorimotor deficits, inc. navigation (sensitivity 95.8%, specificity 91.4%, Cohen’s d=1.89), RT (sensitivity 95.2%, specificity 89.1%, Cohen’s d =1.50) and combined virtual reality modules (sensitivity 95.8%, specificity 96.1%, Cohen’s d =3.59) |
| **Adapted battery of assessments** | Shen J, (2022) (79) | VR-CAT (cognitive battery), Cross Sectional Study, mild to severe TBI (n = 24), orthopaedic controls (n = 30) | Correlation of VR cognitive battery performance findings with National Institute of Health Cognitive battery scores, Feedback on satisfaction and face validity (realism) | | The VR-Cat was rated as adequate for face validity by mTBI (M =3.21, SD =1.41) and the OI (M =3.73, SD =1.11) cohorts, with no significant group differences (p =0.1252). Both cohorts rated high levels of useability and enjoyment.  The composite VR- CAT findings in both cohorts correlated moderately with NIH-toolbox (Overall: r=0.53, p<0.01; orthopedic: r=0.40, p<0.03; mTBI r=0. 57 <0.01). |
|  | Malegiannaki AC, (2024) (80) | Computerized Battery for the Assessment of Attention Disorder (CBAAD) - Battery of Cognitive Assessments, Cross sectional study, TBI (n=10) and Healthy controls (n = 20) | Sensitivity of CBAAD battery screening for attentional dysfunction (AD) in TBI cohort vs controls, Correlation of CBAAD with the Attention Related Cognitive Errors Scale (ARCES) | | Across all CBAAD assessments, the TBI group scored lower than controls (p<0.05) Pearson’s r correlation demonstrated several accuracy and time-based factors correlated with similar ARCES factors (p<.05). |
| **Language and communication** | | | | | |
| **Telemedicine Platform** | | | | | |
| **Discourse assessment** | Turkstra LS (2023) (81) | Aphasia bank™, Mediated Discourse Elicitation Protocol, Internal Telemedicine platform, Cross Sectional Study, repeated measures design - moderate to severe TBI (n =20) | Reliability of discourse assessment (MDEP), when delivered via telemedicine vs in person assessment carried out by separate assessor | | No significant differences found in the telemedicine-based discourse assessment results compared to in-person (P-values on t-tests ranged from 0.08 to 1) |
|  | Cruse N, (2024)(82) | Zoom™ delivered narrative and discourse assessment, cohort study, TBI (n = 20) and Healthy controls (n = 20). | Feasibility assessment (cost, useability, barriers), completion time, Measure of cognition administered via RBANS, Narrative discourse quality (grammar & accuracy analysis) procedural discourse quality (proposition analysis) in TBI vs healthy cohort; interrater reliability. | | Completion time for discourse task was under 10 minutes. Overall cognition TBI group scored lower than control group on assessments of narrative discourse for measures of complete episodes (U=67.50, p<0.001) and missing episode structure (U=122, p=.01), No significant difference seen in the total proposition task (U=172.5, p=.46).  . |
| **Communication assessment** | Rietdijk R, (2017)(83) | Skype™, telemedicine adapted LaTrobe Communication assessment score (LTCA), Cross sectional study, Severe TBI (n = 20) accompanied by family member or carer | Reliability of LTCA (scores the perception of communication skills from the perspective of person with TBI and that of a close other) on telemedicine vs in-person based assessment (performed concurrently) | | There were no significant differences on LTCA scores on telemedicine vs in-person assessments both for TBI subjects themselves (difference=-0.11 [−3.57 to 3.36] P=.95) or significant others (difference =- 1.05 [−4.42 to 2.32] P=.52) on Pearson’s R was 0.871 for TBI cohort and 0.865 for significant others. |
|  | Rietdijk R, (2018)(84) | Skype™, e Adapted Measure of Participation in Conversation (MPC), the Adapted Measure of Support in Conversation (MSC) and the Global Impression scales (GIS), randomized control crossover trial, ABI (n=19) unspecified etiology | Completion by TBI cohort of discourse tasks, delivered both by telemedical and in person. Reliability of findings on AMPCS, AMPC, AMSC, GIS compared to in person | | Skype was used successfully with 17/19 participants to complete discourse tasks No significant differences in assessment findings between telemedicine and in-person assessments, (P>0.05with the exception of GIS “task completion” where telemedicine performance was slightly better (p=.47) |
| **Comprehensive Consultation** | | | | | |
| **Telemedicine** | | | | | |
| **Outpatient Medical Consultation** | Ellis MJ, (2019)(85) | Cisco Jabber based platform, Qualitative study, rural children, (n=20) | Description of service providing telemedicine based mTBI consultation, as well as user feedback & subjects; est. cost-savings vs in-person | | Median time from referral to consultation was 2.0 days, 90% underwent initial consultation via telemedicine (80% exclusively telemedicine). Estimated cost avoidance of $40,972.94 for the entire sample) |
|  | Marckmann C, (2020)(86) | Platform not named, Qualitative study, athletes, mTBI (n=23) and suspected mTBI cohort (n = 184) | Description of provision of telemedicine mTBI clinic, including, wait times, subject satisfaction (Likert scale), estimated cost-savings vs in-person clinic | | Median 1-day wait for consultation post-TBI, mean 54.3 miles travel saved, high program uptake (80% of trainers surveyed used the service); mean satisfaction was 4.75/5 |
|  | Elbin RJ, (2021)(87) | Videoconferencing software not named, Randomized control trial, rural children, mTBI (n =30) | Comparison of mTBI patient and caregiver feedback via therapeutic alliance (TA) survey of telemedicine vs in person delivered mTBI consultation | | Caregivers reported similar levels of therapeutic alliance on in telemedicine vs in-person cohorts. Patient rating of in-person and telehealth TA was similar (t28 = 0.69; P= .49; d= 0.25; 95% CI: −2.34 to 4.74). caregivers rated in-person TA higher than telehealth (U= 52.00; P= .05; d= 0.78; 95% CI, 0.14 to 5.10) |
|  | Caze Ii T, (2020)(88) | Platform not named (internal program), children, mTBI (n=30) | Description of service and subject characteristics and outcomes | | Median time from TBI to 1^st^ visit: 21 days, average of 2.2 visits, 55.6% of subjects cleared to return to learn/play at a median of 15.5 days |

Abbreviations

| ABI | Acquired Brain Injury (May include individuals with TBI and/or other etiologies) |
| --- | --- |
| ANAM | Automated Neuropsychological Assessment Metrics |
| ARCES | Attention-Related Cognitive Errors Test |
| BADS | Behavioral Assessment of the Dysexecutive Syndrome |
| BAM | Brain Acoustic Monitor |
| BAST | Behavioral Assessment Screening Tool |
| BAST-mHealth | Behavioral Assessment Screening Tool for Mobile Health |
| BESS | Balance Error Scoring System |
| BRFSS | Behavioral Risk Factor Surveillance System |
| CATI | Computer Assisted Telephone Interview |
| CATM | Computer assisted text messaging |
| CBB | Cogstate Brief Battery |
| CSS | Concussion Symptom Scale |
| d-NPA | Digital Neuropsychological Assessment |
| DEX | Dysexecutive Questionnaire |
| DMFS | Daily Fatigue Monitoring Scale |
| DMFS | Dutch multifactor fatigue scale |
| EMA | Ecological momentary assessment |
| FAM | Functional Activity Measure |
| FIM | Functional Independence Measure |
| FMA-TV | Telemedicine -adapted Fugyl-Meyer Assessment |
| GOSE | Glasgow Outcome Scale-Extended |
| HRV | Heart Rate Variability |
| HVLT | Hopkins verbal learning test |
| IBPT | Internet-Based Bill-Paying Task |
| ICP | Intracranial Pressure |
| LTCA | La Trobe Communication Assessment |
| mBESS | Modified Balance Error Scoring System |
| ML | Machine Learning |
| MOCA | Montreal Cognitive Assessment |
| MV | Movement Variability |
| NACC-UDS | National Alzheimer’s Coordinating Center Uniform Data Set |
| NIHTB-CB | NIH Toolbox Cognition Battery |
| OMES | Orofacial/Myofunctional Evaluation Scale |
| OSU-BID | Ohio State University Brain Injury Database |
| OSU-TBI-ID | Ohio State University Traumatic Brain Injury Identification Method |
| PANAS | Positive and Negative Affect Schedule |
| PART-O | Participation Assessment with Recombined Tools – Objective |
| PCEI | Post Concussion Excursion Index |
| PCSS | Post-Concussion Symptom Scale |
| PLR | Pupillary Light Reflex |
| PRISMA | Preferred Reporting Items for Systematic Reviews and Meta-Analyse |
| PRQS | Patient-Reported Quality of Sleep |
| RBANS | Repeatable Battery for the Assessment of Neuropsychological Status |
| RFP | Return to field of play |
| RPCS | Rivermead Post-Concussion Symptoms Scale |
| RPQ | Rivermead Post-Concussion Questionnaire |
| SCAT | Sport Concussion Assessment Tool |
| SCAT | Sports Concussion Assessment Tool |
| SHQ | SeaHero Quest |
| SOC | Standard Of Care |
| SOT | Sensory organisation test |
| SRC | Sports related concussion |
| SVFT | Semantic Verbal Fluency Test |
| SWLS | Satisfaction With Life Scale |
| TBI | Traumatic Brain injury |
| TMP | Telemedicine platform |
| VOMS | Vestibular/Ocular Motor Screening |
| WHO | World Health Organization |
|  |  |

Bibliography

1. Falcone M, Yadav N, Poellabauer C, Flynn P. Using isolated vowel sounds for classification of Mild Traumatic Brain Injury. In: 2013 IEEE International Conference on Acoustics, Speech and Signal Processing [Internet]. Vancouver, BC, Canada: IEEE; 2013 [cited 2024 Dec 3]. p. 7577–81. Available from: http://ieeexplore.ieee.org/document/6639136/

2. Yadav N, Poellabauer C, Daudet L, Collins T, McQuillan S, Flynn P. Portable neurological disease assessment using temporal analysis of speech. In: Proceedings of the 6th ACM Conference on Bioinformatics, Computational Biology and Health Informatics [Internet]. Atlanta Georgia: ACM; 2015 [cited 2024 Dec 4]. p. 77–85. Available from: https://dl.acm.org/doi/10.1145/2808719.2808727

3. Shelke S, Agu E. TBI2Vec: Traumatic Brain Injury Smartphone Sensing using AutoEncoder Embeddings. In: 2021 IEEE International Conference on Big Data (Big Data) [Internet]. Orlando, FL, USA: IEEE; 2021 [cited 2024 Dec 3]. p. 4770–9. Available from: https://ieeexplore.ieee.org/document/9671306/

4. Wilkerson GB, Acocello SN, Davis MB, Ramos JM, Rucker AJ, Hogg JA. Wellness Survey Responses and Smartphone App Response Efficiency: Associations With Remote History of Sport-Related Concussion. Percept Mot Skills. 2021 Apr;128(2):714–30.

5. Fischer TD, Red SD, Chuang AZ, Jones EB, McCarthy JJ, Patel SS, et al. Detection of Subtle Cognitive Changes after mTBI Using a Novel Tablet-Based Task. J Neurotrauma. 2016 Jul 1;33(13):1237–46.

6. Yang S, Flores B, Magal R, Harris K, Gross J, Ewbank A, et al. Diagnostic accuracy of tablet-based software for the detection of concussion. Kobeissy FH, editor. PLOS ONE. 2017 Jul 7;12(7):e0179352.

7. Lequerica AH, Lucca C, Chiaravalloti ND, Ward I, Corrigan JD. Feasibility and Preliminary Validation of an Online Version of the Ohio State University Traumatic Brain Injury Identification Method. Arch Phys Med Rehabil. 2018 Sep;99(9):1811–7.

8. Gardner RC, Rivera E, O’Grady M, Doherty C, Yaffe K, Corrigan JD, et al. Screening for Lifetime History of Traumatic Brain Injury Among Older American and Irish Adults at Risk for Dementia: Development and Validation of a Web-Based Survey. J Alzheimers Dis JAD. 2020;74(2):699–711.

9. Sullivan K.A., Caltabiano E. AO - Sullivan KAO https://orcid.org/0000-0002-5952-5114. Online screening assessment of lifetime exposure to traumatic brain injury: a pilot study of associations between exposure and health status. Brain Impair. 2024;25(1):IB23080.

10. Cuthbert JP, Whiteneck GG, Corrigan JD, Bogner J. The Reliability of a Computer-Assisted Telephone Interview Version of the Ohio State University Traumatic Brain Injury Identification Method. J Head Trauma Rehabil. 2016 Jan;31(1):E36–42.

11. Schatz P, Sandel N. Sensitivity and specificity of the online version of ImPACT in high school and collegiate athletes. Am J Sports Med. 2013 Feb;41(2):321–6.

12. Rice V, Boykin G, Alfred P, Lindsay G, Overby C, Jeter A, et al. The investigation of four technologies to assist in detecting mild to moderate traumatic brain injury of U.S. Military service members. Work Read Mass. 2019;63(2):165–80.

13. Schultebraucks K, Yadav V, Galatzer-Levy IR. Utilization of Machine Learning-Based Computer Vision and Voice Analysis to Derive Digital Biomarkers of Cognitive Functioning in Trauma Survivors. Digit Biomark. 2020 Dec 30;5(1):16–23.

14. Sarker P, Hossain KF, Adhanom IB, Pavilionis PK, Murray NG, Tavakkoli A. Analysis of Smooth Pursuit Assessment in Virtual Reality and Concussion Detection using BiLSTM [Internet]. arXiv; 2022 [cited 2024 Dec 4]. Available from: https://arxiv.org/abs/2210.11238

15. Vargas BB, Shepard M, Hentz JG, Kutyreff C, Hershey LG, Starling AJ. Feasibility and accuracy of teleconcussion for acute evaluation of suspected concussion. Neurology. 2017 Apr 18;88(16):1580–3.

16. Sufrinko AM, Howie EK, Charek DB, Elbin RJ, Collins MW, Kontos AP. Mobile Ecological Momentary Assessment of Postconcussion Symptoms and Recovery Outcomes. J Head Trauma Rehabil. 2019 Nov;34(6):E40–8.

17. Liu K, Madrigal E, Chung JS, Broffman JI, Bennett N, Tsai A, et al. Headache Diary Mobile Application for Monitoring and Characterizing Posttraumatic Headaches. J Head Trauma Rehabil. 2022 Jun 1;37(3):171–7.

18. Ezekiel L, Veiga JJD, Ward T, Dawes H, Collett J. Exploring the usability of a smartphone application to monitor fatigue and activity for people with acquired brain injury. Br J Occup Ther. 2023 Nov;86(11):767–76.

19. Ezekiel L, Wilding H, Dearling J, Collett J, Dawes H. Exploring the usefulness of real-time digitally supported fatigue monitoring in fatigue management: Perspectives from occupational therapists and brain injury survivors. Br J Occup Ther. 2024;87(12):783–92.

20. Lazeron-Savu E, Lenaert B, Dijkstra J, Ponds R, van Heugten C. Feasibility of a novel blended-care intervention for fatigue after acquired brain injury: a pilot study of the Tied by Tiredness intervention. Brain Inj. 2024;38(6):448–58.

21. Juengst SB, Graham KM, Pulantara IW, McCue M, Whyte EM, Dicianno BE, et al. Pilot feasibility of an mHealth system for conducting ecological momentary assessment of mood-related symptoms following traumatic brain injury. Brain Inj. 2015;29(11):1351–61.

22. Juengst SB, Terhorst L, Kew CL, Wagner AK. Variability in daily self-reported emotional symptoms and fatigue measured over eight weeks in community dwelling individuals with traumatic brain injury. Brain Inj. 2019 Apr 16;33(5):567–73.

23. Juengst SB, Wright B, Sander AM, Preminger S, Nabasny A, Terhorst L. The Behavioral Assessment Screening Tool for Mobile Health (BASTmHealth): Development and Compliance in 2 Weeks of Daily Reporting in Chronic Traumatic Brain Injury. Arch Phys Med Rehabil. 2023 Feb;104(2):203–10.

24. Little JR, Pavliscsak HH, Cooper MR, Goldstein LA, Fonda SJ. Does Mobile Care ('mCare’) Improve Quality of Life and Treatment Satisfaction Among Service Members Rehabilitating in the Community? Results from a 36-Wk, Randomized Controlled Trial. Mil Med. 2018 Mar 1;183(3–4):e148–56.

25. Forster SD, Gauggel S, Petershofer A, Völzke V, Mainz V. Ecological Momentary Assessment in Patients With an Acquired Brain Injury: A Pilot Study on Compliance and Fluctuations. Front Neurol. 2020 Mar 5;11:115.

26. Rabinowitz A, Hart T, Wilson J. Ecological momentary assessment of affect in context after traumatic brain injury. Rehabil Psychol. 2021 Nov;66(4):442–9.

27. Rabinowitz AR, Hart T. Adherence to high-frequency ecological momentary assessment in persons with moderate-to-severe traumatic brain injury. J Int Neuropsychol Soc. 2024 Oct;30(8):794–8.

28. Sherer M., Juengst S., Sander A.M., Leon-Novelo L., Liu X., Bogaards J., et al. Mood Tracker: A Randomized Controlled Trial of a Self-Monitoring Intervention for Emotional Distress After Traumatic Brain Injury. J Head Trauma Rehabil [Internet]. 2024; Available from: ["https://ezproxy.lib.ucalgary.ca/login?url=https://ovidsp.ovid.com/ovidweb.cgi?T=JS&CSC=Y&NEWS=N&PAGE=fulltext&D=emexa&DO=10.1097%2fHTR.0000000000000945", "https://ucalgary.primo.exlibrisgroup.com/openurl/01UCALG_INST/01UCALG_INST:UCALGARY?sid=OVID:embase&id=pmid:38833719&id=doi:10.1097%2FHTR.0000000000000945&issn=1550-509X&isbn=&volume=40&issue=1&spage=e13&pages=&date=2024&title=The+Journal+of+head+trauma+rehabilitation&atitle=Mood+Tracker%3A+A+Randomized+Controlled+Trial+of+a+Self-Monitoring+Intervention+for+Emotional+Distress+After+Traumatic+Brain+Injury&aulast=Sherer"]

29. Gvozdanovic A, Jozsa F, Fersht N, Grover PJ, Kirby G, Kitchen N, et al. Integration of a personalised mobile health (mHealth) application into the care of patients with brain tumours: proof-of-concept study (IDEAL stage 1). BMJ Surg Interv Health Technol. 2022 Dec;4(1):e000130.

30. Shukla D, Thombre BD, Baby P, Palaninathan J, Subramanian S, Prathyusha P, et al. Validity of Glasgow outcome scale-extended (GOSE) mobile application for assessment of outcome in traumatic brain injury patients. Brain Inj. 2023 Aug 24;37(10):1215–9.

31. Lumetta K, Halama S, Fehr S, Apps J, Thomas D. Functional Assessment of Concussion Tool Application in a Pediatric Concussion Clinic. WMJ Off Publ State Med Soc Wis. 2023 Jul;122(3):191–5.

32. Huber DL, Thomas DG, Danduran M, Meier TB, McCrea MA, Nelson LD. Quantifying Activity Levels After Sport-Related Concussion Using Actigraph and Mobile (mHealth) Technologies. J Athl Train. 2019 Sep;54(9):929–38.

33. Wen PS, Mackey J, Rose D, Waid-Ebbs JK. Smartphone Application for Measuring Community Participation in Veterans With Traumatic Brain Injury. OTJR Occup Ther J Res. 2021 Jul;41(3):196–205.

34. Lee MJ, Gamaldo A, Peters ME, Roy D, Gamaldo CE, Sierra-Arce M, et al. Assessing Sleep Concerns in Individuals With Acquired Brain Injury: The Feasibility of a Smartpad Sleep Tool. J Neuropsychiatry Clin Neurosci. 2021;33(3):225–9.

35. Morrow EL, Mattis-Roesch H, Walsh K, Duff MC. Measurement of Sleep in Chronic Traumatic Brain Injury: Relationship Between Self-report and Actigraphy. J Head Trauma Rehabil. 2024;39(3):E132–40.

36. Nabasny A, Rabinowitz A, Wright B, Wang J, Preminger S, Terhorst L, et al. Neurobehavioral Symptoms and Heart Rate Variability: Feasibility of Remote Collection Using Mobile Health Technology. J Head Trauma Rehabil. 2022 Jun 1;37(3):178–88.

37. Schultz L.S., Murphy M.A., Donegan M., Knights J., Baker J.T., Thompson M.F., et al. Evaluating the Acceptability and Feasibility of Collecting Passive Smartphone Data to Estimate Psychological Functioning in U.S. Service Members and Veterans: A Pilot Study. Mil Med [Internet]. 2024; Available from: ["https://ezproxy.lib.ucalgary.ca/login?url=https://ovidsp.ovid.com/ovidweb.cgi?T=JS&CSC=Y&NEWS=N&PAGE=fulltext&D=emexa&DO=10.1093%2fmilmed%2fusae144", "https://ucalgary.primo.exlibrisgroup.com/openurl/01UCALG_INST/01UCALG_INST:UCALGARY?sid=OVID:embase&id=pmid:38619334&id=doi:10.1093%2Fmilmed%2Fusae144&issn=1930-613X&isbn=&volume=&issue=&spage=&pages=&date=2024&title=Military+medicine&atitle=Evaluating+the+Acceptability+and+Feasibility+of+Collecting+Passive+Smartphone+Data+to+Estimate+Psychological+Functioning+in+U.S.+Service+Members+and+Veterans%3A+A+Pilot+Study&aulast=Schultz"]

38. Suffoletto B, Wagner AK, Arenth PM, Calabria J, Kingsley E, Kristan J, et al. Mobile phone text messaging to assess symptoms after mild traumatic brain injury and provide self-care support: a pilot study. J Head Trauma Rehabil. 2013;28(4):302–12.

39. Anthony CA, Peterson AR. Utilization of a text-messaging robot to assess intraday variation in concussion symptom severity scores. Clin J Sport Med Off J Can Acad Sport Med. 2015 Mar;25(2):149–52.

40. Schoenfeld R, Drendel A, Ahamed SI, Thomas D. Longitudinal Assessment of Acute Concussion Outcomes Through SMS Text (ConText Study). Pediatr Emerg Care. 2022 Jan 1;38(1):e37–42.

41. Karvandi E, Barrett L, Newcombe V, Hutchinson P, Helmy A. Digital health interventions for remote follow-up after mild traumatic brain injury. Br J Neurosurg. 2024;1–7.

42. Shaikh N, Theadom A, Siegert R, Hardaker N, King D, Hume P. Rasch analysis of the Brain Injury Screening Tool (BIST) in mild traumatic brain injury. BMC Neurol. 2021 Dec;21(1):376.

43. Wong AWK, Heinemann AW, Miskovic A, Semik P, Snyder TM. Feasibility of Computerized Adaptive Testing for Collection of Patient-Reported Outcomes After Inpatient Rehabilitation. Arch Phys Med Rehabil. 2014 May;95(5):882–91.

44. Rhea CK, Kuznetsov NA, Ross SE, Long B, Jakiela JT, Bailie JM, et al. Development of a Portable Tool for Screening Neuromotor Sequelae From Repetitive Low-Level Blast Exposure. Mil Med. 2017 Mar;182(S1):147–54.

45. Rhea CK, Yamada M, Kuznetsov NA, Jakiela JT, LoJacono CT, Ross SE, et al. Neuromotor changes in participants with a concussion history can be detected with a custom smartphone app. Roemmich RT, editor. PLOS ONE. 2022 Dec 15;17(12):e0278994.

46. Feigenbaum LA, Kim KJ, Gaunaurd IA, Kaplan LD, Scavo VA, Bennett C, et al. POST-CONCUSSIVE CHANGES IN BALANCE AND POSTURAL STABILITY MEASURED WITH CANESENSE^TM^ AND THE BALANCE ERROR SCORING SYSTEM (BESS) IN DIVISION I COLLEGIATE FOOTBALL PLAYERS: A CASE SERIES. Int J Sports Phys Ther. 2019 Apr;14(2):296–307.

47. Manor B, Zhou J, Lo OY, Zhu H, Gouskova NA, Yu W, et al. Self-Reported Head Trauma Predicts Poor Dual Task Gait in Retired National Football League Players. Ann Neurol. 2020 Jan;87(1):75–83.

48. Kis M. Reliability of a New Test of Balance Function in Healthy and Concussion Populations. J Funct Morphol Kinesiol. 2020 Feb 14;5(1):13.

49. Dummar MK, Crowell MS, Pitt W, Yu AM, McHenry P, Benedict T, et al. The Convergent Validity of the SWAY Balance Application to Assess Postural Stability in Military Cadets Recovering from Concussion. Int J Sports Phys Ther. 2024;19(2):166–75.

50. Tirosh O., Klonis J., Hamilton M., Olver J., Wickramasinghe N., Mckenzie D., et al. Smartphone Technology to Facilitate Remote Postural Balance Assessment in Acute Concussion Management: Pilot Study. Sensors [Internet]. 2024;24(21). Available from: ["https://ezproxy.lib.ucalgary.ca/login?url=https://ovidsp.ovid.com/ovidweb.cgi?T=JS&CSC=Y&NEWS=N&PAGE=fulltext&D=emexb&DO=10.3390%2fs24216870", "https://ucalgary.primo.exlibrisgroup.com/openurl/01UCALG_INST/01UCALG_INST:UCALGARY?sid=OVID:embase&id=pmid:39517769&id=doi:10.3390%2Fs24216870&issn=1424-8220&isbn=&volume=24&issue=21&spage=&pages=&date=2024&title=Sensors+%28Basel%2C+Switzerland%29&atitle=Smartphone+Technology+to+Facilitate+Remote+Postural+Balance+Assessment+in+Acute+Concussion+Management%3A+Pilot+Study&aulast=Tirosh"]

51. Carrick FR, Azzolino SF, Hunfalvay M, Pagnacco G, Oggero E, D’Arcy RCN, et al. The Pupillary Light Reflex as a Biomarker of Concussion. Life Basel Switz. 2021 Oct 18;11(10):1104.

52. McGrath LB, Eaton J, Abecassis IJ, Maxin A, Kelly C, Chesnut RM, et al. Mobile Smartphone-Based Digital Pupillometry Curves in the Diagnosis of Traumatic Brain Injury. Front Neurosci. 2022;16:893711.

53. Maxin AJ, Gulek BG, Lee C, Lim D, Mariakakis A, Levitt MR, et al. Validation of a Smartphone Pupillometry Application in Diagnosing Severe Traumatic Brain Injury. J Neurotrauma. 2023 Oct 1;40(19–20):2118–25.

54. Dutta P. Utility of iPhone-Based Pupillometry in Comparing Pupillary Dynamics Between Sport Concussed Subjects With Photosensitivity and Healthy Controls. J Neuro-Ophthalmol Off J North Am Neuro-Ophthalmol Soc. 2024;44(3):371–5.

55. Mobbs A., Kahn M., Williams G., Mentiplay B.F., Pua Y.-H., Clark R.A. Machine learning for automating subjective clinical assessment of gait impairment in people with acquired brain injury - a comparison of an image extraction and classification system to expert scoring. J NeuroEngineering Rehabil. 2024;21(1):124.

56. Llamas-Ramos R, Llamas-Ramos I, Pérez-Robledo F, Sánchez-González JL, Bermejo-Gil BM, Frutos-Bernal E, et al. Validity of the telematic Fugl Meyer assessment scale – upper extremity (TFMA-UE) Spanish version. Front Neurol. 2023 Aug 10;14:1226192.

57. Zapata-Soria M, Muñoz-Vigueras N, Cabrera-Martos I, López-López L, Ortiz-Rubio A, Valenza MC. Agreement between telerehabilitation and face-to-face orofacial myofunctional assessment in persons with acquired brain injury. Brain Inj. 2023 Jul 3;37(8):669–74.

58. Teel EF, Slobounov SM. Validation of a virtual reality balance module for use in clinical concussion assessment and management. Clin J Sport Med Off J Can Acad Sport Med. 2015 Mar;25(2):144–8.

59. Robitaille N, Jackson PL, Hébert LJ, Mercier C, Bouyer LJ, Fecteau S, et al. A Virtual Reality avatar interaction (VRai) platform to assess residual executive dysfunction in active military personnel with previous mild traumatic brain injury: proof of concept. Disabil Rehabil Assist Technol. 2017 Oct;12(7):758–64.

60. Painter DR, Norwood MF, Marsh CH, Hine T, Harvie D, Libera M, et al. Immersive virtual reality gameplay detects visuospatial atypicality, including unilateral spatial neglect, following brain injury: a pilot study. J NeuroEngineering Rehabil. 2023 Nov 23;20(1):161.

61. Rebchuk AD, Alimohammadi A, Yuan M, Cairncross M, Torres IJ, Silverberg ND, et al. Assessment of Prorated Scoring of an Abbreviated Protocol for the National Institutes of Health Toolbox Cognition Battery. J Int Neuropsychol Soc JINS. 2020 Nov;26(10):1045–50.

62. Spreij LA, Gosselt IK, Visser-Meily JMA, Nijboer TCW. Digital neuropsychological assessment: Feasibility and applicability in patients with acquired brain injury. J Clin Exp Neuropsychol. 2020 Sep 13;42(8):781–93.

63. Spreij LA, Gosselt IK, Visser-Meily JMA, Hoogerbrugge AJ, Kootstra TM, Nijboer TCW. The journey is just as important as the destination-Digital neuropsychological assessment provides performance stability measures in patients with acquired brain injury. PloS One. 2021;16(7):e0249886.

64. Wallace SE, Donoso Brown EV, Schreiber JB, Diehl S, Kinney J, Zangara L. Touchscreen tablet-based cognitive assessment versus paper-based assessments for traumatic brain injury. NeuroRehabilitation. 2019;45(1):25–36.

65. Chen PM, Lee S, Cruz LD, Lopez M, Thomas A, Chen JW, et al. iPad-Based Neurocognitive Testing (ImPACT-QT) in Acute Adult Mild Traumatic Brain Injury/Concussion: Study on Practicality and Bedside Cognitive Scores in a Level-1 Trauma Center. Am Surg. 2024;90(6):1570–6.

66. Mendez-Lopez M., Juan M.-C., Burgos T., Mendez M., Fidalgo C. AO - Fidalgo CO https://orcid.org/0000-0002-4732-6417. How people with brain injury run and evaluate a SLAM-based smartphone augmented reality application to assess object-location memory. PsyCh J. 2024;13(6):903 EP – 914.

67. Morrow EL, Nelson LA, Duff MC, Mayberry LS. An Ecological Momentary Assessment and Intervention Tool for Memory in Chronic Traumatic Brain Injury: Development and Usability of Memory Ecological Momentary Intervention. JMIR Rehabil Assist Technol. 2024;11(101703412):e59630.

68. Canty AL, Fleming J, Patterson F, Green HJ, Man D, Shum DHK. Evaluation of a virtual reality prospective memory task for use with individuals with severe traumatic brain injury. Neuropsychol Rehabil. 2014;24(2):238–65.

69. Goverover Y, DeLuca J. Actual reality: Using the Internet to assess everyday functioning after traumatic brain injury. Brain Inj. 2015;29(6):715–21.

70. Martínez-Pernía D, Núñez-Huasaf J, Del Blanco Á, Ruiz-Tagle A, Velásquez J, Gomez M, et al. Using game authoring platforms to develop screen-based simulated functional assessments in persons with executive dysfunction following traumatic brain injury. J Biomed Inform. 2017 Oct;74:71–84.

71. VanNostrand M, Rieger B, Baracks J, Neville C. Development and initial validation of a digital divided-attention neurocognitive test for use in concussion assessment. Brain Inj. 2019 Jun 7;33(7):941–51.

72. Nadler Tzadok Y, Eliav R, Portnoy S, Rand D. Establishing the Validity of the Internet-Based Bill-Paying Task to Assess Executive Function Deficits Among Adults With Traumatic Brain Injury. Am J Occup Ther Off Publ Am Occup Ther Assoc. 2022 Jul 1;76(4):7604205110.

73. Lencses A, Mikula B, Mioni G, Rendell PG, Denes Z, Demeter G. Prospective memory functions in traumatic brain injury: The role of neuropsychological deficits, metamemory and impaired self-awareness. J Neuropsychol [Internet]. 2024;(101468753). Available from: ["https://ezproxy.lib.ucalgary.ca/login?url=https://ovidsp.ovid.com/ovidweb.cgi?T=JS&CSC=Y&NEWS=N&PAGE=fulltext&D=medp&DO=10.1111%2fjnp.12388", "https://ucalgary.primo.exlibrisgroup.com/openurl/01UCALG_INST/01UCALG_INST:UCALGARY?sid=OVID:medline&id=pmid:39188167&id=doi:10.1111%2Fjnp.12388&issn=1748-6645&isbn=&volume=&issue=&spage=&pages=&date=2024&title=Journal+of+Neuropsychology&atitle=Prospective+memory+functions+in+traumatic+brain+injury%3A+The+role+of+neuropsychological+deficits%2C+metamemory+and+impaired+self-awareness.&aulast=Lencses"]

74. Del Giovane M, Trender W, Bălăeţ M, Mallas EJ, Jolly A, Bourke NJ, et al. Online Cognitive Assessment of Traumatic Brain Injury Patients is Accessible, Feasible and Performs Well Relative to Established Clinical [Internet]. SSRN; 2023 [cited 2024 Dec 3]. Available from: https://www.ssrn.com/abstract=4352554

75. Pellinen J., Sillau S., Morrison C., Maruff P., O’Brien T.J., Penovich P., et al. Engagement in online cognitive testing with the Cogstate brief battery among a multinational cohort of people with focal epilepsy. Epilepsy Behav. 2024;159:109953.

76. Seton C, Coutrot A, Hornberger M, Spiers HJ, Knight R, Whyatt C. Wayfinding and path integration deficits detected using a virtual reality mobile app in patients with traumatic brain injury. Cristofori I, editor. PLOS ONE. 2023 Mar 9;18(3):e0282255.

77. S Okahashi, H Mizumoto, K Ueno, M Yokoyama, Nagano A, K Seki, et al. Assessment of convalescent brain-damaged patients using a virtual shopping test with different task difficulties. In Japan; 2014.

78. Teel E, Gay M, Johnson B, Slobounov S. Determining sensitivity/specificity of virtual reality-based neuropsychological tool for detecting residual abnormalities following sport-related concussion. Neuropsychology. 2016 May;30(4):474–83.

79. Shen J, Koterba C, Samora J, Leonard J, Li R, Shi J, et al. Usability and validity of a virtual reality cognitive assessment tool for pediatric traumatic brain injury. Rehabil Psychol. 2022 Nov;67(4):587–96.

80. Malegiannaki AC, Garefalaki E, Pellas N, Kosmidis MH. Virtual Reality Assessment of Attention Deficits in Traumatic Brain Injury: Effectiveness and Ecological Validity. Multimodal Technol Interact. 2024 Jan 3;8(1):3.

81. Turkstra LS, Quinn-Padron M, Johnson JE, Workinger MS, Antoniotti N. In-Person Versus Telehealth Assessment of Discourse Ability in Adults With Traumatic Brain Injury. J Head Trauma Rehabil. 2012 Nov;27(6):424–32.

82. Cruse N, Piotto V, Coelho C, Behn N. Telehealth administration of narrative and procedural discourse: A UK and US comparison of traumatic brain injury and matched controls. Int J Lang Commun Disord. 2024 Mar;59(2):519–31.

83. Rietdijk R, Power E, Brunner M, Togher L. Reliability of Videoconferencing Administration of a Communication Questionnaire to People With Traumatic Brain Injury and Their Close Others. J Head Trauma Rehabil. 2017;32(6):E38–44.

84. Rietdijk R, Power E, Brunner M, Togher L. The reliability of evaluating conversations between people with traumatic brain injury and their communication partners via videoconferencing. Neuropsychol Rehabil. 2020 Jul 2;30(6):1074–91.

85. Ellis MJ, Boles S, Derksen V, Dawyduk B, Amadu A, Stelmack K, et al. Evaluation of a pilot paediatric concussion telemedicine programme for northern communities in Manitoba. Int J Circumpolar Health. 2019 Jan 1;78(1):1573163.

86. Marckmann C, John D. Telemedicine, Quality Initiative for Concussion Management. J Nurse Pract. 2020 Jan;16(1):e9–12.

87. Elbin RJ, Stephenson K, Lipinski D, Maxey K, Womble MN, Reynolds E, et al. In-Person Versus Telehealth for Concussion Clinical Care in Adolescents: A Pilot Study of Therapeutic Alliance and Patient Satisfaction. J Head Trauma Rehabil. 2022 Aug 1;37(4):213–9.

88. Caze Ii T, Knell GP, Abt J, Burkhart SO. Management and Treatment of Concussions via Tele-Concussion in a Pediatric Setting: Methodological Approach and Descriptive Analysis. JMIR Pediatr Parent. 2020 Aug 13;3(2):e19924.
